# Fel d 1‐Expressing Plant‐Derived Bioparticle: A Novel Treatment for Cat Allergy

**DOI:** 10.1111/all.70280

**Published:** 2026-03-19

**Authors:** Janice A. Layhadi, Liliana Cifuentes Gutierrez, Sean T. Keane, William Fulton, Nichell A. Samson, Lily Y. D. Wu, Paulina Filipaviciute, Prista Hikmawati, Oleksandra Fedina, Ana Jimenez‐Gil, Stephen R. Durham, Guy Scadding, Guy Tropper, Louis‐Philippe Vézina, Patrick Colin, Ronald van Ree, Mohamed H. Shamji

**Affiliations:** ^1^ National Heart and Lung Institute Imperial College London London UK; ^2^ Royal Brompton Hospital, Guy's and St Thomas' NHS Foundation Trust London UK; ^3^ Angany Inc. Lévis Quebec Canada; ^4^ Department of Experimental Immunology, Amsterdam UMC University of Amsterdam Amsterdam the Netherlands; ^5^ Amsterdam Institute for Infection and Immunity Inflammatory Diseases Amsterdam the Netherlands

**Keywords:** allergen immunotherapy, allergy, cat allergy, tolerance induction

## Abstract

**Background:**

Allergen immunotherapy (AIT) is the only disease‐modifying therapeutic approach for cat allergy, though it requires at least three years of treatment and can potentially induce severe systemic reactions. Plant‐derived bioparticles expressing Fel d 1 allergen (Fel d 1 *e*BP) have been developed as a novel therapeutic candidate for cat allergy. We aimed to investigate the allergenicity and immunogenicity profile of Fel d 1 *e*BP.

**Methods:**

Fel d 1 *e*BP was synthesised in vivo in *Nicotiana benthamiana* and confirmed by cryo‐electron microscopy and tomography. Immune modulatory properties of purified natural Fel d 1 (nFel d 1) and Fel d 1 *e*BP were assessed at T and B cells by flow cytometry in 12 cat‐allergic subjects (CA) and 12 non‐atopic controls (NAC). Single‐cell RNA‐seq was used to assess molecular mechanisms of Fel d 1 *e*BP immune skewing. The safety of Fel d 1 *e*BP was assessed by measuring basophil responsiveness in whole blood and further confirmed in vivo by its administration as a skin prick test (SPT) in 20 cat‐allergic individuals.

**Results:**

Fel d 1 *e*BP was shown to be a strong inducer of Th1 cells (*p* < 0.05) and IL‐10^+^ non‐Th2 cells (*p* < 0.05) in CA subjects. Fel d 1 *e*BP showed a stronger trend for inducing IL‐10^+^ Breg cells compared to nFel d 1, peaking at 3 μg/mL. scRNA‐seq analyses demonstrated that Fel d 1 *e*BP targets the induction of protective metallothionein genes, CCL18^+^ monocytes and naïve B cells that are interferon responsive and metabolically activated. Moreover, Fel d 1 *e*BP demonstrated reduced capacity to elicit basophil activation (*p* < 0.001) and histamine release (*p* < 0.01), indicating their hypoallergenic nature. Administration of titrated doses of Fel d 1 *e*BP through skin prick test revealed that they are well‐tolerated with reduced mean wheal compared to native Fel d 1 in an open‐label Phase 0 study (all, *p* < 0.001).

**Conclusions:**

We demonstrate that Fel d 1 *e*BP is hypoallergenic and demonstrates tolerogenic properties, making it a novel candidate for use in AIT for cat allergy.

## Graphical abstract

This study investigates the allergenicity and immunogenicity profile of a novel plant‐derived Fel d 1 bioparticle (*e*BP). Fel d 1 eBP promotes immune tolerance by redirecting allergic Th2 responses toward Th1 and IL‐10–producing regulatory pathways, including Bregs. Its reduced allergenicity and targeted modulation of innate and B‐cell responses were confirmed by molecular profiling and reduced skin and basophil reactivity in cat‐allergic subjects. Abbreviations: BAT, basophil activation test; Breg, regulatory B cell; CA, cat‐allergic subjects; CCL18, chemokine (C‐C motif) ligand 18; DAO, diamine oxidase; *e*BP, enveloped bioparticle; EC_50_ , half maximal effective concentration; IFN, interferon; IFNAR, interferon alpha/beta receptor; IL‐10, interleukin 10; MT, metallothionein; NAC, non‐atopic control; scRNAseq, single‐cell RNA sequencing; SPT, skin prick test; Th2, T helper cell type 2; Th1, T helper cell type 1.

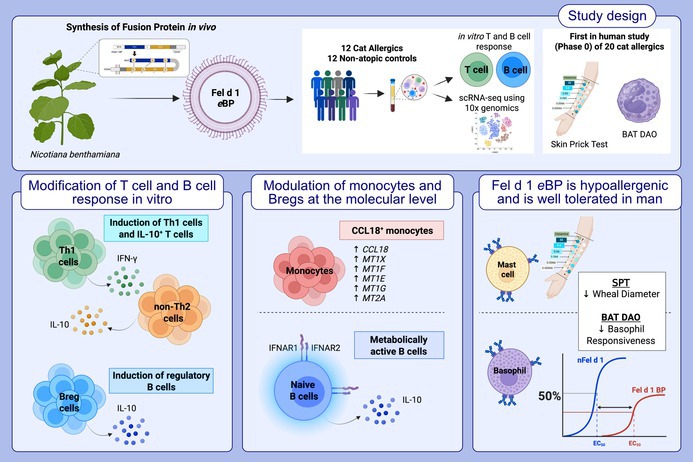

## 1 Introduction

Sensitisation to cats is one of the most common causes of allergic diseases, affecting an estimated 10%–20% of the global population [1, 2, 3, 4, 5, 6]. Symptoms of cat allergy vary considerably between individuals, from rhinoconjunctivitis to potentially life‐threatening asthma exacerbations, with some studies demonstrating sensitisation to cat being a strong risk factor for asthma [7, 8]. Eight cat allergens are currently recognised by the World Health Organization (WHO), and *Felis domesticus* allergen 1 (Fel d 1) is the major and most potent allergen [9], with approximately 90% of cat‐allergic individuals being sensitised toward Fel d 1 [10]. As with other allergies, the common recommendation of symptom management is avoidance and reduced exposure to cat allergens, though this can be challenging for most individuals. Another common method of management includes the use of common pharmacotherapies (such as anti‐histamine, corticosteroids, etc.), which provide temporary symptom relief and can manage symptoms in some, but not all, sufferers. Allergen immunotherapy (AIT) is indicated in those who do not respond to common pharmacotherapies, but is also aimed at the broader community who suffer from allergy, with or without asthma.

When administered for at least 3 years, AIT remains the only disease‐modifying treatment with proven clinical benefit upon discontinuation of the treatment. Mechanisms underlying tolerance induction following AIT have been studied extensively, and involvement of various immune responses has been indicated, such as modulation of T and B cell responses [11, 12, 13, 14] and induction of blocking antibodies, such as IgG_4_ [15, 16, 17], that can compete with IgE to inhibit subsequent activation of basophil and mast cells to dampen the early phase allergic response. Both forms of AIT, subcutaneous (SCIT) or sublingual (SLIT), have been recommended for the management of cat allergies, though existing studies and clinical trials have provided conflicting results [18, 19, 20, 21]. To this day, AIT using conventional allergens is still associated with a low compliance rate due to the long‐term regimen and risk of severe allergic reactions. More recently, the use of recombinant‐blocking monoclonal IgG_4_ against Fel d 1 in cat‐allergic individuals has also been explored and has demonstrated suppression of IgE binding to Fel d 1 [22, 23], modulation of immune responses and a reduction in clinical symptoms lasting up to 84 days, essentially providing symptomatic relief. However, these data are preliminary and further studies are warranted to identify the ideal treatment for cat allergy, which comprises a therapy that is shorter in duration, with superior safety and immunogenic profile.

A promising approach in allergen immunotherapy (AIT) involves the use of bioparticles (BPs) that display repetitive conformational epitopes, effectively eliciting robust T and B cell responses. Among these, viral‐like particles (VLPs) have been successfully utilised in several approved vaccines, such as those for Hepatitis B (Recombivax HB by Merck & Co. and Engerix‐B by GlaxoSmithKline) [24, 25], human papillomavirus (Gardasil by Merck & Co. and Cervarix by GlaxoSmithKline) [26], and Hepatitis E (Hecolin by Innovax) [27], demonstrating their proven efficacy and safety. Furthermore, VLP‐based vaccines are also being developed for allergic diseases [28], including food allergies, with encouraging early‐stage results. In this study, we introduce a novel AIT platform based on plant‐derived enveloped bioparticles (*e*BPs) displaying Fel d 1, a major cat allergen. Unlike traditional VLPs, which are virus‐derived, these fully plant‐based *e*BPs are inherently non‐viral, potentially offering an improved safety profile. Building upon prior in vitro evidence suggesting hypoallergenicity, we confirmed reduced allergenicity using basophils from cat‐allergic donors and validated these findings with in vivo titrated skin prick tests. Moreover, Fel d 1 *e*BPs exhibited notable anti‐inflammatory effects and modulated Th2 responses in peripheral blood mononuclear cells (PBMCs). These immune‐modulating effects were supported by flow cytometry and single‐cell RNA sequencing, which revealed distinct immune‐deviation mechanisms and indications of potential tolerogenic responses.

## 2 Methods

### 2.1 Fel d 1 Enveloped Bioparticle Manufacturing

Fel d 1 *e*BP was manufactured by Angany Inc. following internal procedures as described earlier for Der p 2 *e*BP [29, 30]. A positive control of purified nFel d 1 (Inbio, Cardiff, UK) was used in all in vitro assays. Quantification of Fel d 1 was done by immunoblot. nFel d 1 and Fel d 1 *e*BP were randomised and blinded for all in vitro assays, and all operators remain blinded until the completion of analysis.

### 2.2 Subject Recruitment for Mechanistic Study

Patients with cat allergy (CA; *n* = 12) and non‐atopic controls (NACs; *n* = 12) provided blood samples and answered clinical symptom questionnaires (Table 1). The study was approved by the South West London REC3 Research Ethics Committee and the Research Office of the Royal Brompton and Harefield NHS Foundation Trust. An informed consent form was collected from the participants. Inclusion and exclusion criteria can be found in the online supplementary method.

**TABLE 1:** Patient demographics for mechanistic, pre‐clinical study.

	CA (*n* = 12)	NAC (*n* = 12)
Sex (F/M)	6/6	7/5
Age (y), mean (range)	35.25 (22:58)	37 (25:55)
Duration of cat dander‐induced allergic rhinoconjunctivitis (y), mean ± SD	14.08 ± 14.02	0.00 ± 0.00
Visual Analogue Score (VAS) score, mean ± SD	55.00 ± 24.74	0.00 ± 0.00
Cat dander SPT (mm), mean ± SD	8.33 ± 2.42	0.00 ± 0.00
Cat dander‐specific IgE (kU_A_/L), mean ± SD	3.2 ± 3.73	0.00 ± 0.00
Fel d 1‐specific IgE (kU_A_/L), mean ± SD	2.87 ± 2.73	0.00 ± 0.01
Total IgE (kU_A_/L), mean ± SD	154.30 ± 177.20	16.21 ± 17.84
Asthma (%, n)	25% (3)	0% (0)
Monosensitised patients (n)	1	0
Polysensitised patients (n)	11	0

Abbreviations: CA: Cat‐allergic subjects; F: Female; M: Male; NAC: Non‐atopic controls; n: Number of patients; SD: Standard deviation; SPT: Skin prick test; y: Year.

### 2.3 Clinical Trial Study Design

An open‐label Phase 0 study (REC reference [10]/SC/0464) was conducted to test the safety and allergenicity of the Fel d 1 *e*BP in 21 cat‐allergic individuals with positive skin prick test (wheal diameter ≥ 7 mm), and cat dander and Fel d 1‐specific IgE ≥ 1 kU_A_/L (Table 2 and Table S1). Safety and tolerability were assessed through skin prick test and intradermal testing. The early phase skin response (wheal diameter) to Fel d 1 *e*BP in a titrated SPT compared to a commercial cat dander allergen extract (ALK‐Abello Cat Soluprick 100,000 SQ/ml containing a mixture of cat allergen components, including Fel d 1) was evaluated, with tests done in duplicate, using both arms. The primary outcome was the provocation concentration of allergen that causes a > 5 mm skin wheal. The provocation concentration of allergen that caused a > 3 mm skin wheal was determined as a secondary endpoint. In addition to the early phase response, the late phase skin response following intradermal administration of Fel d 1 *e*BP was compared to the commercial extract at 6.5 h post‐injection, using 1:1000 and 1:100 dilutions of the concentrations of Fel d 1 *e*BP and commercial cat dander extract, which elicited a > 3 mm wheal, respectively. Ex vivo allergenicity was assessed through the basophil activation test. Clinical Trial Inclusion and Exclusion Criteria can be found in the online supplementary method.

**TABLE 2:** Patient demographics for clinical trial study at baseline.

	CA (*n* = 20)
Age (y), mean (range)	31.10 (21:59)
Cat dander first SPT (mm), mean ± SD	8.90 ± 1.74
Cat dander second SPT (mm), mean ± SD	8.48 ± 1.52
Cat dander‐specific IgE (kU_A_/L), mean ± SD	15.62 ± 26.17
Fel d 1‐specific IgE (kU_A_/L), mean ± SD	11.25 ± 16.76
Total IgE (kU_A_/L), mean ± SD	305.01 ± 283.93
Asthma (%, n)	35% (7)
Monosensitised patients (n)	0
Polysensitised patients (n)	20

Abbreviations: CA: Cat‐allergic subjects; n: Number of patients; SD: Standard deviation; SPT: Skin prick test; y: Year.

### 2.4 Cellular Analyses

Methods outlining basophil activation test, IgE‐FAB assay, peripheral blood mononuclear cells (PBMCs), in vitro T‐ and B‐cell stimulation and unbiased clustering analyses are outlined in the Supporting Information section.

### 2.5 Single Cell RNAseq Using 10× Genomics

Live PBMCs were cultured with nFel d 1 or Fel d 1 *e*BP for 6 days at 37°C and 5% CO_2_. After 6 days of stimulation, PBMCs were washed, counted using trypan blue exclusion, and immediately processed for single‐cell 5′ chemistry (10× Genomics). Single‐cell suspensions were loaded onto a Chromium Single Cell chip and prepared using the Chromium Single Cell 5′ v2 Reagent Kit (10× Genomics) according to the manufacturer's instructions to allow encapsulation with barcoded Gel beads at a target capture rate of approximately 10,000 individual cells per sample. Further details can be found in the Supporting Information section.

### 2.6 Statistical Analysis

Between‐group and within‐group comparisons were performed by the Mann–Whitney U test and the Wilcoxon matched‐pairs signed‐rank test, respectively. Correlation analysis was determined using the Spearman rank method. The statistical software package used was GraphPad Prism (version 10; GraphPad Software). A *p*‐value of less than 0.05 was considered significant. Statistical analysis for single‐cell immune profiling was performed using ANOVA. Filtration criteria to identify genes differentially expressed between groups were performed based on a *p*‐value of less than 0.05.

## 3 Results

### 3.1 Biosynthesis and Purification of Fel d 1 *e*BP

**FIGURE 1:**
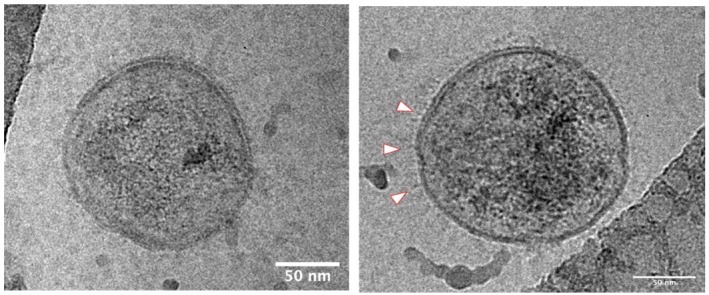
Construction of Fel d 1 *e*BPs. Cryo‐EM visualisation demonstrated the bioparticles to be spherical proteolipidic structures of about 100–150 nm. Presence of hairy structures at the surface of the supramolecular structures is highlighted by arrowheads (right panel). Scale bars are 50 nm.

The Fel d 1 *e*BP, consisting of a fusion of the CH1 and CH2 polypeptides, previously described for soluble recombinant Fel d 1 [31], is synthesised in vivo in *Nicotiana Benthamiana* [30]. Fel d 1 *e*BP was characterised by cryo‐electron microscopy and tomography. The two methods indicated that Fel d 1 *e*BPs consist of a lipid‐bilayer membranous body, the surface of which is covered with protein clusters with a transmembrane domain. Based on Cryo‐EM and tomography, Fel d 1 *e*BP appears as spherical particles with an average diameter of 100–150 nm and may be hollow, as no polymeric material or structures can be seen in their lumen (Figure 1).

### 3.2 Modulation of T Cell Responses by Fel d 1 *e*BP

**FIGURE 2:**
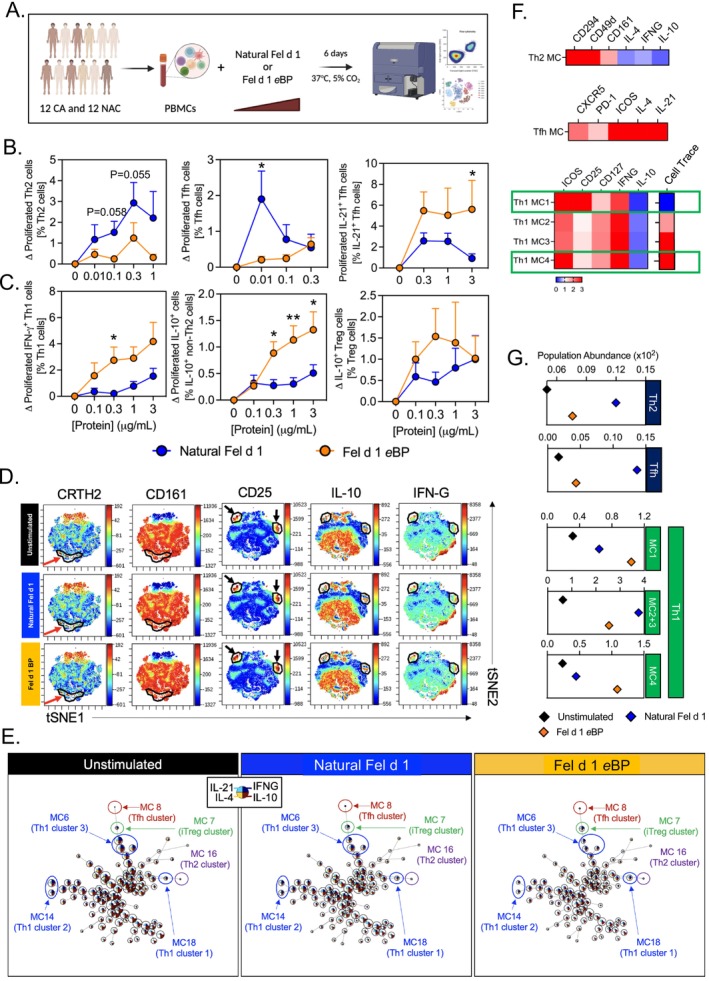
Fel d 1 *e*BP demonstrates a capacity to modulate the T cell responses. (A) Schematic representation of the workflow. (B, C) Effect of response to increasing doses of nFel d 1 and Fel d 1 *e*BP in vitro in CA (*n* = 12) subjects on proliferation of *(B)* T_H_2 (CD4^+^, CD27^−^, CRTH2^+^), Tfh cells (CD4^+^, CXCR5^+^, PD‐1^+^), IL‐21^+^ Tfh cells (IL‐21^+^, CD4^+^, CXCR5^+^, PD‐1^+^) and *(C)* T_H_1 (CD4^+^, IFN𝛄^+^), IL10^+^ non‐T_H_2 (CD4^+^, CRTH2^−^, IL10^+^), and IL10^+^ Treg (CD4^+^, CD25^+^, CD127^lo^, IL10^+^) cells. (D) Unbiased clustering analysis using viSNE was performed on flow cytometry data generated in vitro to identify expression of T‐cell subsets in response to no stimulation, nFel d 1 (0.3 μg/mL) or Fel d 1 *e*BP (0.3 μg/mL) stimulation. (E) FlowSOM analysis illustrating metaclusters affected by no stimulation, stimulation, nFel d 1 (0.3 μg/mL) or Fel d 1 *e*BP (0.3 μg/mL) stimulation as a star plot. (F) Heat map illustrating the phenotype of CD4^+^ T cells. (G) Effect of no stimulation, nFel d 1 and Fel d 1 *e*BP stimulation on population abundance of several metaclusters. Between‐group comparison statistical analysis was performed by the Mann–Whitney U test; *p < 0.05, **p < 0.01, ***p < 0.001. Data are shown as means ± SEMs.

The effect of nFel d 1 and Fel d 1 *e*BP on T cell responses was investigated in an in vitro study with cat‐allergic (CA) subjects (Figure 2A). Their impact on Th2, Tfh cells, IL‐21^+^ Tfh cells and other subsets like Th1, IL‐10^+^ non‐Th2 cells, and IL‐10^+^ Tregs was evaluated.

In CA subjects, nFel d 1 induced a dose‐dependent proliferation of Th2 and Tfh cells (Figure 2B). Fel d 1 *e*BP showed a trend toward reduced Th2 proliferation (*p* = 0.058 at 0.1 μg/mL and *p* = 0.055 at 0.3 μg/mL), with a significant reduction in Tfh proliferation at 0.01 μg/mL (*p* < 0.05). Fel d 1 *e*BP also demonstrated a dose‐dependent induction of proliferation of IL‐21+ Tfh cells reaching significance at 3 μg/mL (*p* < 0.05), and induced significant proliferation of IFN‐γ^+^ Th1 cells (*p* < 0.05) and IL‐10^+^ non‐Th2 cells (*p* < 0.05) compared to nFel d 1 (Figure 2C). No significant changes were seen in IL‐10^+^ Tregs (Figure 2C, right panel). In NAC subjects, Fel d 1 *e*BP showed similar trends but without significance (Figure S1A,B).

Unbiased clustering using viSNE and FlowSOM confirmed these effects. viSNE revealed distinct Th2, inducible Treg (iTreg), and Th1 cell populations (Figure 2D). FlowSOM analysis identified distinct clusters for Th1, Tfh, and Th2 cells (Figure 2E,F). Fel d 1 *e*BP reduced Th2 and Tfh clusters while specifically targeting two Th1 clusters: A proliferative Th1 cluster (Th1 MC1) and a non‐proliferative Th1 cluster (Th1 MC4) (Figure 2G).

### 3.3 Fel d 1 *e*BP Targets the Induction of B Regulatory Cells

**FIGURE 3:**
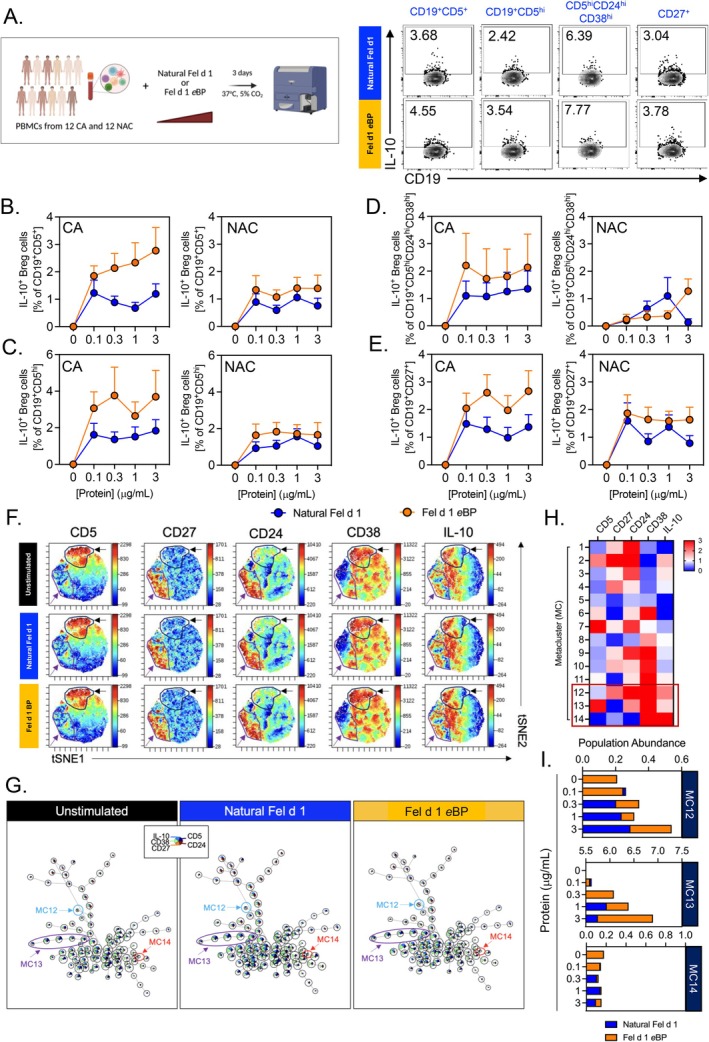
Fel d 1 *e*BP has the capacity to induce subsets of IL‐10‐producing Breg cells. (A) Schematic representation of the experimental workflow (left) and flow cytometry representative plots of the induction of IL‐10^+^CD19^+^CD5^+^ (CD5^+^), IL10^+^CD19^+^CD5^hi^ (CD5^hi^), IL10^+^CD19^+^CD5^hi^CD24^hi^CD38^hi^ (CD24^hi^CD38^hi^), and IL10^+^CD19^+^CD27^+^ (CD27^+^) Breg cells stimulated with nFel d 1 or Fel d 1 bioparticle (right). (B–E) Graphical representation of the induction of *(B)* IL‐10^+^CD19^+^CD5^+^, *(C)* IL10^+^CD19^+^CD5^hi^, *(D)* IL10^+^CD19^+^CD5^hi^CD24^hi^CD38^hi^ and *(E)* IL10^+^CD19^+^CD27^+^ stimulated with nFel d 1 or Fel d 1 *e*BP for CA (*n* = 12) and NAC (*n* = 11) subjects. (F) Unbiased clustering analysis using viSNE was performed on flow cytometry data generated in vitro to identify expression of Breg subsets in response to no stimulation, nFel d 1 (0.3 μg/mL) or Fel d 1 *e*BP (0.3 μg/mL) stimulation. (G) FlowSOM analysis illustrating metaclusters affected by no stimulation, stimulation, nFel d 1 (0.3 μg/mL) or Fel d 1 *e*BP (0.3 μg/mL) stimulation as a star plot. (H) Heat map illustrating phenotype of CD19^+^ B cells within each metacluster. (I) Effect of no stimulation, nFel d 1 and Fel d 1 *e*BP stimulation on population abundance of metaclusters (MC) 12, 13 and 14. Between‐group comparison statistical analysis was performed by the Mann–Whitney U test; *p < 0.05, **p < 0.01, ***p < 0.001. Data are shown as means ± SEMs.

Regulatory B (Breg) cells, known for their IL‐10 production, play a key role in immune tolerance during successful AIT. The ability of Fel d 1 *e*BP to induce IL‐10^+^ Breg cells was investigated, focusing on four subsets: (1) IL‐10^+^CD19^+^CD5^+^, (2) IL‐10^+^CD19^+^CD5^hi^, (3) IL‐10^+^CD19^+^CD5^hi^CD24^hi^CD38^hi^, and (4) IL‐10^+^CD19^+^CD27^+^ (Figure 3A).

In cat‐allergic (CA) individuals, Fel d 1 *e*BP showed a stronger trend for inducing IL‐10^+^ Breg cells compared to nFel d 1, peaking at 3 μg/mL (Figure 3B–E; left panel). This effect was absent in non‐allergic controls (NAC) (Figure 3B–E; right panel). Similar trends were observed in activated, transitional, and memory Breg cells, though without statistical significance.

Unbiased clustering via viSNE and FlowSOM identified two distinct IL‐10^+^ Breg populations and three phenotypically different meta‐clusters (MC12, MC13, and MC14) (Figure 3F–H). Quantification confirmed that Fel d 1 *e*BP induced MC12 and MC13 Breg clusters compared to nFel d 1 (Figure 3I), supporting its role in modulating tolerogenic Breg responses.

### 3.4 Proportion of Natural Treg Cells Is Not Modulated by Fel d 1 *e*BP

Like Breg cells, the induction of natural Treg cells (FOXP3^+^ Treg cells) has also been demonstrated as a strong indication of tolerance induction, with functional Treg cells (denoted with a loss of SATB1 expression) playing specific roles in dampening Th2 responses. In this study, the capacity of Fel d 1 *e*BP to elicit natural Treg cells was investigated in 12 CA and 12 NAC, and enumeration of different subsets of Treg cells was performed on the flow cytometry (Figure S2A).

In both CA and NAC subjects, nFel d 1 elicited a dose‐dependent increase in the proportion of FOXP3^+^ natural (Figure S2B), memory (Figure S2C), and naïve (Figure S2D) T reg cells, with peak responses that vary within the subsets. Interestingly, in all subsets of Treg cells, Fel d 1 *e*BP elicited an overlapping response with no further ability to enhance the proportion of circulating Treg cells compared to nFel d 1. In parallel to this, the functional counterparts of each Treg subset investigated, which are denoted by a lack of SATB1 expression, were also investigated. Both nFel d 1 extract and Fel d 1 *e*BP had a similar capacity to induce a dose‐dependent increase in the level of functional natural Treg (Figure S2E), functional memory (Figure S2F), and functional naïve (Figure S2G) Treg cells. These findings highlight that natural Treg cells are not targeted by Fel d 1 *e*BP in their mechanism of action.

### 3.5 Fel d 1 *e*BP Induces Its Immune‐Modifying Properties by Modulating Genes Belonging to the Metallothionine Family

**FIGURE 4:**
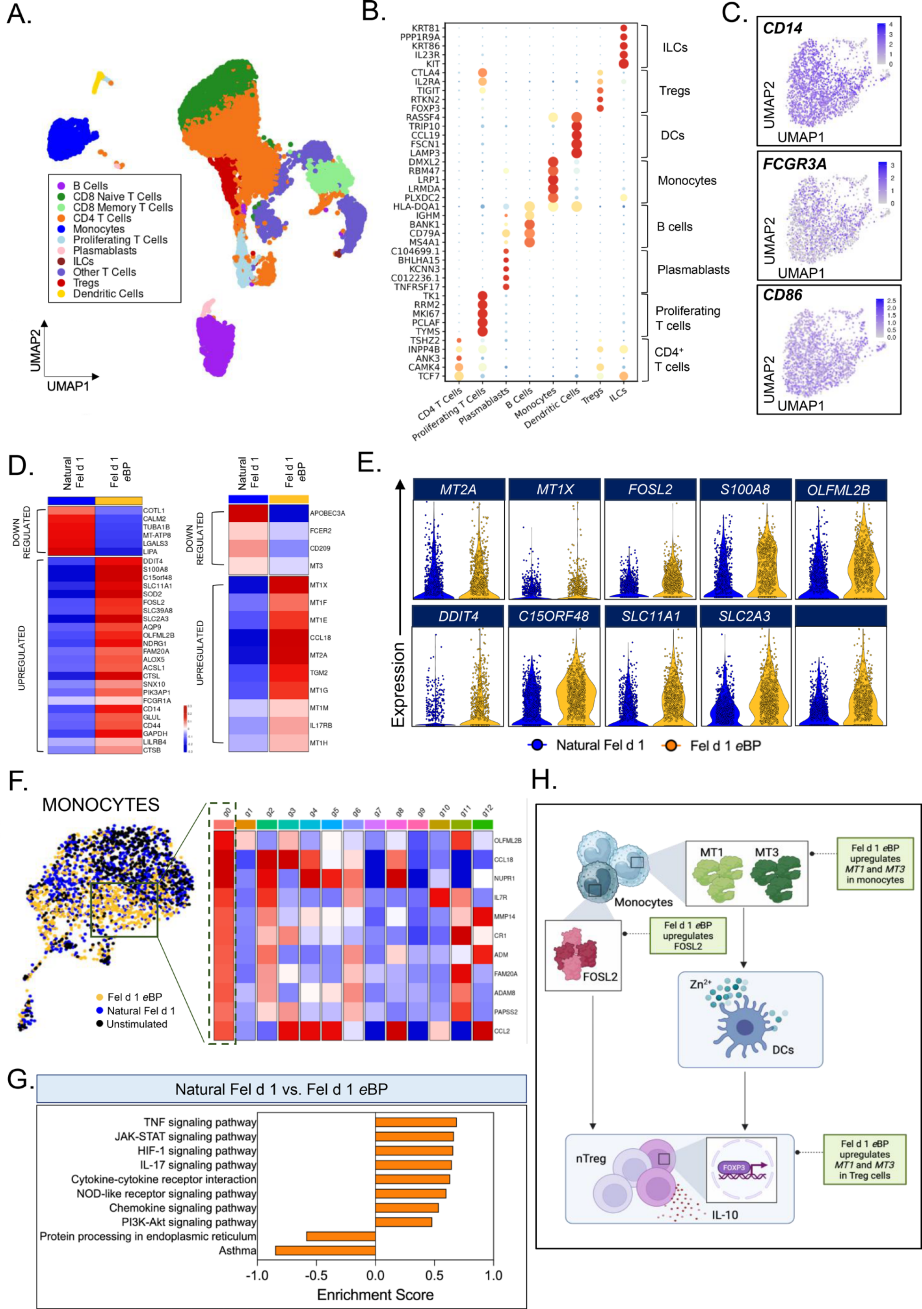
Fel d 1 *e*BP is capable of modulating metallothionine‐associated genes in monocytes. (A) UMAP of cell identities and clusters identified by unsupervised clustering after 6 days in vitro culture of PBMCs from cat‐allergic subjects (*n* = 3). (B) Heatmap of the top 5 differentially expressed gene markers for each cell subset reported (*p* < 0.05, LFC > 0.5). (C) Expression of monocyte markers. (D) Heatmap of top down‐ and up‐regulated genes (left panel) and genes associated with the metallothionine family (right panel) following stimulation with nFel d 1 and Fel d 1 *e*BP. (E) Violin plots highlighting differential gene expression in monocytes following stimulation with nFel d 1 or Fel d 1 *e*BP. (F) UMAP of monocyte population stratified by stimulating conditions (unstimulated—black, nFel d 1—blue and Fel d 1 *e*BP). (G) Pathway enrichment analyses on gene changes using the KEGG database in monocytes. (H) Genes associated with metallothionine activation and signalling.

Single‐cell RNA‐seq was performed on PBMCs from three cat‐allergic individuals after 6‐day stimulation with either nFel d 1 or Fel d 1 *e*BP. A total of 28,702 high‐quality cells were analysed, revealing 12 annotated clusters, including ILCs, dendritic cells, B cells (including plasmablasts), and T cell subsets (CD4, CD8, Tregs, proliferating T) (Figure 4A,B). Further analyses focused on monocytes, Tregs, and B cells.

Monocytes (*n* = 2,206) expressed *CD14, FCGR3A*, and *CD86* (Figure 4C). nFel d 1 upregulated pro‐allergic genes (e.g., *IL17RB, FCER2, CCL18*), while Fel d 1 *e*BP significantly modulated 250 (vs. Fel d 1) and 527 (vs. unstimulated) genes (Adj. *p* < 0.05, LFC = 0.5) (Figure S3), including tolerogenic markers like *DDIT4, NMES1, FOSL2, MT1X*, and *MT2A* (Figure 4D,E). Unbiased clustering identified 12 monocyte subsets (Figure S4A,B), including a unique population induced solely by Fel d 1 *e*BP, characterised by high *CCL18*, *IL7R*, *OLFML2B*, and *NUPR1* expression (Figure 4F). KEGG analysis revealed distinct enrichment of immune regulatory pathways (TNF, JAK–STAT, IL‐17, chemokine signalling) in response to Fel d 1 *e*BP (Figure 4G,H).

**FIGURE 5:**
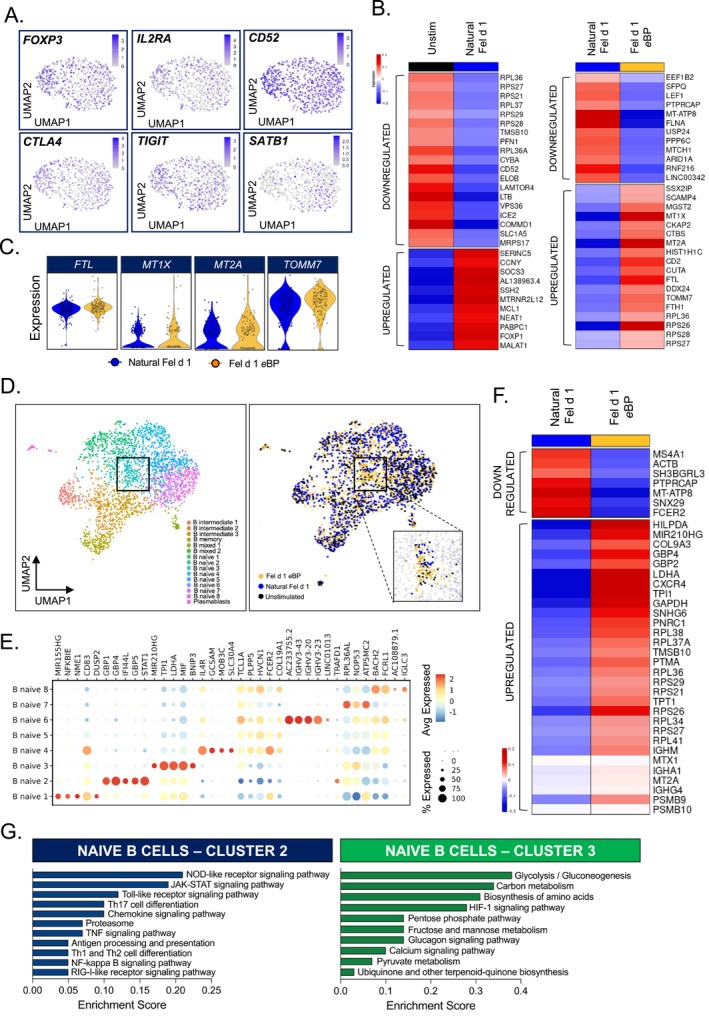
Fel d 1 *e*BP can modulate molecular mechanisms associated with regulatory T and B cells. (A) Expression of Treg markers. (B) Heatmap of top down‐ and up‐regulated genes (left panel) and genes associated with the metallothionein family (right panel) following stimulation with nFel d 1 and Fel d 1 *e*BP. (C) Violin plots highlighting differential gene expression in Treg cells following stimulation with nFel d 1 or Fel d 1 *e*BP. (D) UMAP of different B cell subsets identified by unsupervised clustering (left panel) and identified by stimulating conditions (right panel). (E) Dot plot of the top five differentially expressed gene markers for each B naïve cell subset (*p* < 0.05, LFC > 0.5). (F) Heatmap of top down‐ and up‐regulated genes in naïve B cell population following stimulation with native Fel d 1 or Fel d 1 *e*BP. (G) Pathway enrichment analyses on gene changes using the KEGG database in cluster 2 (left panel) and cluster 3 (right panel) of naïve B cells.

Additionally, the effect of Fel d 1 *e*BP on Treg cells (*n* = 636) was also investigated. Whilst we previously reported that Fel d 1 *e*BP did not modulate levels of natural FOXP3^+^ Tregs, we did observe modulation at the molecular level. Treg cells displayed high expression of *FOXP3, IL2RA, CTLA4*, and *TIGIT* (Figure 5A) and showed modest transcriptional changes following Fel d 1 stimulation (10 DEGs). In contrast, Fel d 1 *e*BP induced broader transcriptional modulation (17 vs. unstimulated, 6 vs. Fel d 1), upregulating tolerogenic genes *ICOS, MT1X, and MT2A* (Figure 5B,C). Reactome pathway analysis indicated enrichment in antigen presentation, MHC complex, and humoral response pathways, alongside suppression of IL‐4 response and modulation of type II IFN signalling (Figure S6). These findings suggest that Fel d 1 *e*BP enhances tolerogenic programs in monocytes and Tregs via induction of metallothionein family genes.

### 3.6 In vitro Stimulation With Fel d 1 *e*BP Preferentially Induces Activated Naïve B Cells

Unsupervised clustering identified 15 B cell clusters, with 8 clusters of naïve B cells (1,522 cells). Stimulation with Fel d 1 *e*BP predominantly targeted Cluster 3 of naïve B cells (Figure 5D,E and Figure S5). Differential gene analysis revealed 250 DEGs in naïve B cells (Adj. *p* < 0.05, LFC = 0.5), including downregulation of *ACTB, MS4A1, FCER2*, and upregulation of interferon (*GBP2, GBP4*) and metabolic genes (*TPI1, LDHA, PSMB9*) (Figure 5F). Further analysis of Cluster 2 (98 cells) and Cluster 3 (143 cells) revealed that Cluster 2 is likely IFN‐responsive, while Cluster 3 resembles metabolically active naïve B cells. Pathway enrichment showed distinct profiles: Cluster 2 was enriched in immune signalling (e.g., NOD‐like, JAK–STAT, TNF), while Cluster 3 was enriched in metabolic pathways (e.g., glycolysis, pyruvate metabolism) (Figure 5G). These findings suggest Fel d 1 *e*BP specifically targets B cell subsets that may mediate suppressive effects.

### 3.7 Fel d 1 *e*BP Is Hypoallergenic and Has Reduced Capacity to Induce FcεRI‐ and FcεRII‐Mediated Allergic Responses

**FIGURE 6:**
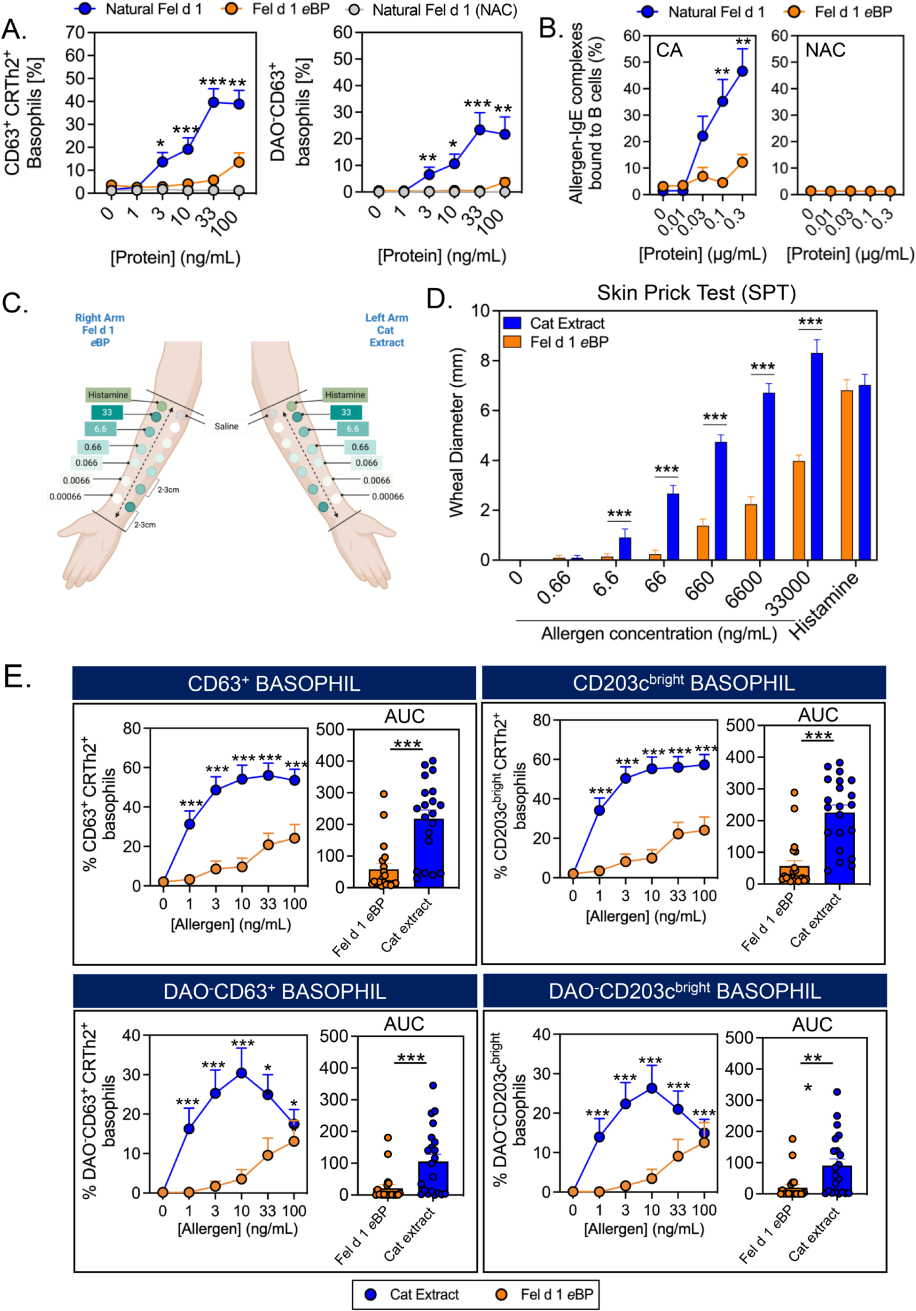
Fel d 1 *e*BP demonstrated its tolerability and hypoallergenic profile through basophil activation test and in vivo skin prick test administration. (A) Effect of nFel d 1 and Fel d 1 bioparticle on basophil activation (CD63^+^) and histamine release (DAO^−^, CD63^+^) of CA subjects (*n* = 12) and the effect of nFel d 1 on basophil activation of NAC subjects (*n* = 12). (B) Percentage of allergen‐IgE binding inhibition of CA subjects (*n* = 12) and NAC subjects (*n* = 12) serum treated with varying concentrations of either natural Fel d1 or Fel d 1 *e*BP. (C) Schematic diagram illustrating administration of incremental doses of nFel d 1 (Left arm) or Fel d 1 *e*BP (Right arm) in duplicate bidirectionally as a skin prick test (SPT) with positive histamine and negative saline controls. Doses specified in the diagram represent Fel d 1 content in μg/mL. (D) SPT Wheal diameter measurements in response to incremental doses of nFel d 1 or Fel d 1 *e*BP in cat‐allergic subjects (*n* = 21). (E) Proportion of basophil activation (CD63^+^ and CD203c^bright^) and histamine release (DAO^−^CD63^+^ and DAO^−^CD203c^bright^) in response to increasing doses of nFel d 1 or Fel d 1 *e*BP.

The ability of Fel d 1 *e*BP to trigger FcεRI and FcεRII‐mediated allergic responses was assessed through basophil activation and IgE‐facilitated allergen binding assays. Basophil activation was measured by CD63/CD203c expression and histamine release (DAO). Multi‐parametric flow cytometry showed that nFel d 1 elicited dose‐dependent basophil activation in CA but not NAC individuals, with peak responses at 33 ng/mL (Figure 6A, Figure S7A,B). In contrast, Fel d 1 *e*BP shifted the dose–response curve to the right and induced significantly lower basophil activation (*p* < 0.001 for CD63^+^, *p* < 0.05 for CD203c^bright^) (Figure S7C,D).

Histamine release assays showed that nFel d 1 induced dose‐dependent release with peak responses at 33 ng/mL in CA but not NAC subjects, while Fel d 1 *e*BP failed to induce histamine release up to 100 ng/mL (*p* < 0.01) Figure S7C,D. The IgE‐FAB assay demonstrated that nFel d 1 formed dose‐dependent IgE complexes binding to B cells in CA, but Fel d 1 *e*BP did not bind to CD23 on B cells at any concentration (Figure 6B). These results confirm the hypoallergenic nature of Fel d 1 *e*BP in mediating FcεRI and FcεRII responses.

### 3.8 Fel d 1 *e*BP Is Well‐Tolerated Following in Vivo Administration in Cat‐Allergic Subjects

To evaluate the safety and tolerability of Fel d 1 *e*BP, a skin prick test (SPT) comparing escalating doses of Fel d 1 *e*BP and nFel d 1 (from commercial cat dander extract) was conducted in 20 cat‐allergic individuals (Figure 6C). Fel d 1 *e*BP induced significantly smaller wheals at all concentrations (*p* < 0.001), showing > 50‐fold reduced allergenicity (Figure 6D, Table S2). Intradermal tests produced similar early and late phase responses, despite using 23‐fold higher Fel d 1 *e*BP concentrations due to its lower SPT reactivity. Both tests were well tolerated with no serious adverse events.

Basophil activation tests confirmed hypoallergenicity. nFel d 1 triggered dose‐dependent activation (CD63^+^, CD203c^bright^ basophils), while Fel d 1 *e*BP showed a rightward dose–response shift and significantly lower AUC (*p* < 0.001) (Figure 6E, top panel). Similarly, histamine release assays showed reduced activation by Fel d 1 *e*BP (*p* < 0.001) (Figure 6E, bottom panel). These findings support the clinical tolerability and hypoallergenic nature of Fel d 1 *e*BP.

## 4 Discussion

The identification of an ideal candidate for use in AIT to treat allergic asthma (AA) and allergic rhinitis (AR) has been of great interest for both scientists and clinicians. Novel approaches that can allow greater safety with less severe side effects, along with superior immunogenicity, remain the main criteria of novel AIT strategies. One such approach includes the use of bioparticles, such as the *e*BP, for the presentation of target antigen to the immune system, as they can elicit a strong T and B cell immune response. Here, we investigated the suitability of Fel d 1 *e*BP as a novel strategy for AIT to treat cat allergy.

The allergenicity of Fel d 1 *e*BP was evaluated based on its ability to trigger FcεRI‐ and FcεRII‐mediated allergic responses. Compared to nFel d 1, Fel d 1 *e*BP was significantly less potent, requiring 45–50 times higher concentrations to induce basophil activation and histamine release in cat‐allergic individuals. This hypoallergenic profile aligns with findings from other allergy therapies, such as peptide‐based or modified allergen extracts, which show reduced degranulation and histamine release [22, 29, 30, 32, 33]. Our results support the use of Fel d 1 *e*BP as a safe candidate for cat allergy treatment, further evidenced by its inability to form IgE complexes or engage CD23 on B cells—features consistent with a tolerogenic immune response typical of successful allergen immunotherapy. Importantly, its reduced allergenic potency suggests potential for use without the need for updosing [17, 22].

Capsid VLPs have been previously explored as delivery systems for AIT [34, 35] but showed limited success due to side effects [36, 37, 38]. In contrast, enveloped VLPs (eVLPs), which retain host cell membrane components, offer immunostimulatory benefits and are considered self‐adjuvanted [39]. These self‐assembling eVLPs have shown promise in other disease models, such as influenza, where viral capsid proteins are fused with target antigens in various expression systems [40, 41]. Recently, a plant‐based *e*BP platform free of viral proteins was developed to reduce potential side effects. This system successfully displayed allergens like Der p 2 on *Nicotiana benthamiana*‐derived particles, inducing a Th1/Treg‐skewed immune response [42, 43]. It has also been reported that the design and production pipeline allows the expression of approximately 1000 allergen trimers on the surface of the *e*BP. It is previously hypothesised that the structural constraints of the *e*BP meant that only a very limited number of the allergen molecules per *e*BP can bind to the same mast cells, limiting the number of crosslinking events and subsequent activation and degranulation of these effector cells [30]. Furthermore, the role of membrane‐bound glucosylceramides in NKT‐cell interactions is also under investigation. Der p 2 *e*BPs have been linked to strong IgG responses, particularly IgG2a [29, 30]. Using this platform, Fel d 1‐expressing *e*BPs elicited a strong IFN‐γ^+^ Th1 and IL‐10^+^ non‐Th2 response in cat‐allergic individuals, while failing to activate pro‐inflammatory Th2, Th2A, and Tfh cells. These cells drive allergic responses by producing IL‐4, IL‐5, IL‐13, and IL‐21, promoting IgE class switching and sustaining inflammation [44, 45, 46]. The ability of Fel d 1 *e*BP to suppress all three pro‐allergic T cell subsets highlights its strong potential as a candidate for AIT [45, 47].

The induction of regulatory B and T cells has been widely reported following successful AIT [48, 49, 50, 51, 52]. Bregs play a key role in immune tolerance by producing IL‐10, suppressing Th2 cells, inducing Tregs, and generating IgG_4_‐blocking antibodies [48, 49, 50, 51, 52, 53, 54, 55, 56]. In our study, Fel d 1 *e*BP promoted the expansion of IL‐10^+^ Breg cells, particularly within a subset of metabolically activated naïve B cells. Metabolic pathways are fundamental to both normal and pathogenic immune processes. B cells dynamically adjust their metabolic activity in response to environmental cues and functional demands. While naïve B cells are metabolically quiescent, activation through the B‐cell receptor or co‐receptor signalling triggers engagement of pathways such as glycolysis, fatty acid oxidation, oxidative phosphorylation and the pentose phosphate pathway [57, 58, 59, 60, 61]. In this study, Fel d 1 *e*BP selectively induced several of these metabolic programs within a distinct cluster (cluster 3) of naïve B cells. Specifically, the upregulation of glycolysis and HIF‐1 signalling observed in Cluster 3—including high expression of the glycolytic *LDHA* gene—has previously been linked to IL‐10 production in B cells [60, 62]. Although these naïve B cells did not themselves express IL‐10 transcripts, the observed in vitro induction of IL10^+^ Bregs suggests a potential mechanism through which Fel d 1 *e*BP may exert its hypoallergenic properties. To substantiate this mechanism, Bregs, characterised by the capacity to produce IL‐10, contribute to IgG_4_ production by suppressing inflammation and modulating B cell differentiation. Given the direct impact of Fel d 1 *e*BP on Breg induction both in vitro and at the single‐cell model, it is likely that its immunomodulatory effects involve enhanced production of IgG_4_‐blocking antibodies. These can inhibit allergen‐IgE binding by reducing cross‐linking and promoting immune tolerance via Fab‐arm exchange.

In monocytes, Fel d 1 *e*BP induced the upregulation of *C15orf48 (NMES1*), a marker of type 1 dendritic cells, and *FOSL2*, a regulator involved in *FOXP3* expression and B cell differentiation. Elevated FOSL2 expression has been linked to reduced Tfh differentiation and attenuation of allergic inflammation in murine models [63, 64, 65]. scRNA‐seq analysis revealed that Fel d 1 *e*BP also strongly induced metallothionein genes (MT1X, MT2A), which are involved in cellular homeostasis and have been shown to prevent allergic asthma in mice [66]. These genes are also markers of phenotypically regulatory, though not necessarily functional, DCs and Tregs. This suggests that Fel d 1 *e*BP may promote, at the molecular level, a tolerogenic phenotype in monocytes and Tregs, though functional validation is still required.

Our study provides early evidence supporting the use of Fel d 1 *e*BP in AIT for cat allergy. In a Phase 0 safety and allergenicity trial, Fel d 1 *e*BP was well‐tolerated by cat‐allergic individuals, with only moderate skin reactions at the highest concentrations. Ex vivo, it showed hypoallergenic properties, including reduced basophil activation and degranulation. Several treatment strategies for cat allergy have shown varying success. A recent approach using Fel d 1 on Qβ‐VLPs demonstrated reduced mast cell activation, though less effective than Fel d 1 *e*BP [67]. Monoclonal antibody therapies, such as REGN1908–1909, have shown promise by blocking early allergic responses through a single dose administration [22, 23]. A Phase 2 trial further showed prevention of early asthmatic responses, though sustained efficacy may require dosing every 2–3 months [68, 69]. Another candidate, Tezepelumab combined with AIT, reduced TNSS by 36% at treatment end and by 24% a year later in the CATNIP Phase 1/2 trial, though long‐term maintenance has not been examined [70]. Recombinant hypoallergenic vaccines [71, 72, 73, 74] and T‐cell‐epitope‐based peptide therapies [75] aim to reduce IgE‐mediated side effects while maintaining or enhancing T‐cell tolerance induction. Similarly, Fel d 1‐based fusion vaccines, including intralymphatic formulations, have demonstrated that targeted allergen presentation can accelerate tolerance development with fewer administrations [76]. The Fel d 1 *e*BP platform builds upon these concepts by providing a molecularly defined system that sequesters allergen epitopes and modulates B‐ and T‐cell responses, thereby offering a potentially safer and more precise approach to achieve long‐term immune tolerance.

Findings from this study provide early cellular, molecular, and clinical evidence of the potential application of Fel d 1 *e*BP in the treatment of cat allergy. Our data indicate that the response is not strictly unidirectional but involves elements of both regulatory and inflammatory signalling, which reflects the biological complexity of the system. It is essential to highlight the study's limitations. Firstly, it was not possible to obtain a non‐allergen‐expressing bioparticle as a control due to production challenges. In‐planta budding of bioparticles depends on the presence of virus‐mimicking sequences attached to the allergen within the construct. In the absence of an allergen, bioparticle budding is not achievable. Secondly, whilst previous studies using the same bioparticle have shown induction of blocking IgG antibodies, we have not measured Fel d 1‐specific IgG levels in our study. It would be noteworthy to measure these blocking antibodies in future clinical trial studies of Fel d 1 *e*BP. Thirdly, while AIT is known to induce immune deviation toward Th1/IgG responses, its disease‐modifying effects likely involve additional mechanisms, including suppression of Th2/Th2A inflammation and induction of regulatory T and B cells. Finally, because several of the transcriptional changes we observed have not been previously reported, the available literature for comparison is limited; therefore, our interpretations in these novel areas are necessarily data‐driven and should be viewed in the context of current knowledge boundaries. Together, these processes may underpin the establishment of a tolerant state, though our study does not directly assess long‐term tolerance. To remain cautious in our interpretation, we describe our findings primarily in terms of suppression of pro‐inflammatory responses, induction of regulatory cell activity, and immune deviation toward Th1 responses, which collectively may contribute to tolerance.

Whilst there is still an ongoing search for the ideal candidate for cat allergy treatment, novel strategies using Fel d 1 *e*BP used in our study provide early mechanistic evidence underscoring their immune‐deviation and likely tolerogenic role as strong contenders for the future of cat allergy. A phase 1b trial in cat‐allergic patients with asthma with or without rhinitis is planned for 2026.

## Author Contributions

J.A.L., S.T.K., N.S., L.Y.D.W., P.F., P.H., and G.T. performed experiments and analyses of data; W.F. did bioinformatic analyses of single‐cell data. G.S., L.C.G., O.F., A.J.‐G., and S.R.D. recruited clinical samples for the study and performed a Phase 0 clinical trial. J.A.L., M.H.S., R.R. and L.‐P.V. designed the mechanistic study, wrote the manuscript, and interpreted the data. G.S., S.R.D., and P.C. designed the clinical study. All authors critically read the manuscript.

## Funding

This work was supported by Angany Inc., Canada.

## Conflicts of Interest

G.S., S.K., L.C.G., W.F., N.S., L.Y.D.W., P.F., P.H., O.F., A.J.‐G. report declare no conflicts of interest. J.A.L. reports grants via Biomedical Research Funding (Imperial College BRC), all outside the submitted work; M.H.S. reports research grants from Immune Tolerance Network, Medical Research Council, Allergy Therapeutics, LETI Laboratorios, Rovolo Biotherapeutics and lecture fees from Allergy Therapeutics and LETI Laboratorios, all outside the submitted work. R.v.R. Consultancies for HAL Allergy BV, Citeq BV, Angany Inc., Reacta Healthcare Ltd., Mission MightyMe, The Protein Brewery and AB Enzymes GmbH. Speaker fees from Thermo Fisher Scientific, ALK and HAL Allergy. Stock options from Angany Inc. V.G., L.‐P.V. are former or current employees of Angany Inc.

## Supporting information


**Data S1:** Supporting Information.

**Figure S1** Fel d 1 *e*BP lacks the capacity to modulate T cell responses in non‐atopic controls. (A, B) Effect of response to increasing doses of natural Fel d 1 and Fel d 1 *e*BP in vitro in CA (*n* = 12) subjects on proliferation of *(A)* T_H_2 (CD4^+^, CD27^−^, CRTH2^+^), T_H_2A (CD4^+^, CD27^−^, CRTH2^+^, CD161^+^, CD49d^+^), Tfh cells (CD4^+^, CXCR5^+^, PD‐1^+^) and *(B)* T_H_1 (CD4^+^, IFN𝛄^+^), IL10^+^ non‐T_H_2 (CD4^+^, CRTH2^−^, IL10^+^), and IL10^+^ Treg (CD4^+^, CD25^+^, CD127^lo^, IL10^+^) cells. Between‐group comparison statistical analysis was performed by the Mann–Whitney U test; *p < 0.05, **p < 0.01, ***p < 0.001. Data are shown as means ± SEMs.

**Figure S2** Fel d 1 *e*BP lacks the capacity to induce Natural, Memory and Naïve Treg subsets. (A) Flow cytometry representative plots of Treg (CD4^+^CD25^hi^CD127^lo^) cell response to no stimulation, natural Fel d 1 or Fel d 1 *e*BP stimulation in CA and NAC subjects. (B) Effect of natural Fel d 1 and Fel d 1 *e*BP on Natural (CD4^+^FOXP3^+^CD25^hi^CD127^lo^), Memory (CD4^+^CD45RO^+^FOXP3^+^CD25^hi^CD127^lo^), Naïve (CD4^+^CD45RO^−^FOXP3^+^CD25^hi^ CD127^lo^) Treg cells of CA subjects (*n* = 12) and NAC subjects (*n* = 10). (C) Effect of natural Fel d 1 and Fel d 1 *e*BP on functional Natural (SATB1^−^FOXP3^+^CD25^hi^CD127^lo^), Memory (SATB1^−^CD45RO^+^FOXP3^+^CD25^hi^CD127^lo^), Naïve (SATB1^−^CD45RO^−^FOXP3^+^CD25^hi^CD127^lo^) Treg subsets of CA subjects (*n* = 12) and NAC subjects (*n* = 10). Between‐group comparison statistical analysis was performed by the Mann–Whitney U test; *p < 0.05, **p < 0.01, ***p < 0.001. Data are shown as means ± SEMs.

**Figure S3** Differential gene expression analysis of monocytes treated with natural Fel d 1, Fel d 1 *e*BP and untreated. (A) Volcano plot depiction and (B) heatmap representation of the top 30 differentially expressed genes by adjusted *p*‐value between natural Fel d 1 and Fel d 1 *e*BP (Left), unstimulated and natural Fel d 1 (middle), and unstimulated and Fel d 1 BP‐treated PBMCs performed using scRNAseq following 6 days of in vitro stimulation. Statistical analyses were performed using ANOVA, with *p* < 0.05 denoting a differential expression.

**Figure S4** Single‐cell RNA sequencing reveals 13 distinct clusters of monocytes. (A) UMAP showing 13 distinct clusters of monocytes from PBMCs following 6 days of in vitro stimulation with natural Fel d 1, Fel d 1 *e*BP and unstimulated from 3 cat‐allergic individuals. (B) Heatmap depicting the expression of the top 5 genes in each of the monocyte clusters labelled g0–g12.

**Figure S5** Differential abundance testing of scRNAseq data identifies a Fel d 1 *e*BP targeted neighbourhoods of naïve B cells. (A, B) Beeswarm plots depicting the differential abundance of different neighbourhoods within the B cell subclusters and (B) highlighted neighbourhoods targeted by the Fel d 1 *e*BP (*p* ‐adjusted< 0.05).

**Figure S6** Gene Ontology GSEA Pathway analysis of Treg cells reveals a dampening of type 2 inflammatory signalling pathway in the presence of Fel d 1 *e*BP. Schematic representation of the clustering of gene ontology pathways determined by GSEA pathway enrichment analysis of Fel d 1 *e*BP‐treated Treg cells compared to natural Fel d 1, with highlighted pathways of interest and the associated genes and corresponding normalised enrichment score (NES; NES = +1.6, *p* < 0.2 and NES = −1.76, *p* < 0.2).

**Figure S7**Fel d 1 *e*BP demonstrates hypoallergenic characteristics in vitro. (A) Flow cytometry representative plots of basophil activation and histamine release after ex vivo natural Fel d 1 or Fel d 1 *e*BP stimulation. (B–D) Effect of natural Fel d 1 and Fel d 1 *e*BP on *(B)* basophil activation (CD203c^bright^) of CA subjects (*n* = 12) and the effect of natural Fel d 1 on basophil activation of NAC subjects (*n* = 12), and *(C)* the corresponding AUC analysis of basophil activation and histamine release after natural Fel d1 and Fel d 1 *e*BP stimulation of CA subjects (*n* = 12). (D) Table demonstrating the AUC of basophil activation and histamine release.

**Table S1** Baseline skin wheal diameter statistics.
**Table S2** Mean post‐SPT wheal size following ANG101 and ALK Soluprick administration. Highlighted values are associated with patients showing a minimal 5 mm wheal diameter for both products.

## Data Availability

The data that support the findings of this study are available from the corresponding author upon reasonable request.
