# Supplementary material for: Fel d 1‐Expressing Plant‐Derived Bioparticle: A Novel Treatment for Cat Allergy
Source: Allergy. 2026 Mar 19;81(6):2156–71. doi: 10.1111/all.70280 (PMC13256289; doi:10.1111/all.70280)
Supplement: Supplementary file 1 — Data S1: Supporting Information. [file ALL-81-2156-s008.docx]

**ONLINE SUPPLEMENTARY**

**Fel d 1-expressing plant-derived bioparticle: A novel treatment for cat allergy**

Janice A. Layhadi^1^*, Liliana Cifuentes Gutierrez^1,2^* Sean T. Keane^1^, William Fulton^1^, Nichell Samson^1^, Lily Y.D. Wu^1^, Paulina Filipaviciute^1^, Prista Hikmawati^1^, Gabija Drazsdauskaite^1^, Oleksandra Fedina^1,2^, Ana Jimenez-Gil^1,2^, Stephen R. Durham^1,2^, Guy Scadding^1^^,2^, Guy Tropper^3^, Louis‐Philippe Vézina^3^, Patrick Colin^3^, Ronald van Ree^4,5^ and Mohamed H. Shamji^1^

^1^ National Heart and Lung Institute, Imperial College London, London SW7 2AZ, United Kingdom

^2^ Royal Brompton Hospital, Guy’s and St Thomas’ NHS Foundation Trust, London

^3^ Angany Inc., Lévis, QC, Canada

^4^ Department of Experimental Immunology, Amsterdam UMC Location University of Amsterdam, Amsterdam, The Netherlands

^5^ Amsterdam Institute for Infection and Immunity, Inflammatory Diseases, Amsterdam, The Netherlands

*Authors with equal contribution

**Corresponding author:**

Professor Mohamed H. Shamji

Immunomodulation and Tolerance Group, Allergy & Clinical Immunology,

Inflammation, Repair and Development, National Heart and Lung Institute,

Sir Alexander Fleming Building, Imperial College London,

South Kensington Campus, London SW7 2AZ, United Kingdom

Tel: +44 020 7594 3476

E-mail: [m.shamji@imperial.ac.uk](mailto:m.shamji@imperial.ac.uk)

**Subjects**

Inclusion criteria for cat allergic individuals include a positive specific IgE level of > 0.35 kU_A_/L to Fel d 1 measured using ImmunoCAP (Thermo Fisher Scientific, Waltham Mass), and a positive skin prick test (wheal ≥ 3mm in diameter) to cat dander allergen extract (ALK-Abelló). Non-atopic patients were recruited based on a negative skin prick test specific IgE to Fel d 1. Informed consent form was collected from the participants. Patients were excluded from the study if they had undergone immunotherapy treatment to cat dander allergen in the past 5 years, had any previous exposure to monoclonal antibody treatment, or had undergone any antihistamine or decongestion therapy within 7 days prior to the initial screening for the study. Patients that have a history of chronic immune diseases or medical conditions (other than asthma, atopic dermatitis or allergic rhinitis) were also excluded from the study.

**Clinical Trial Inclusion and Exclusion Criteria**

Adults aged 18 - 60 years of age with minimum of 2 years of cat dander induced moderate to servere persistent allergic rhinitis or rhinoconjunctivitis with or without allergic asthma (Global Initiatve for Asthma (GINA) < Step 3 and FEV-1 > 80% at screening), with a positive SPT for cat dander (mean wheal diameter ≥ 7mm) and Cat and Fel d1 – specific IgE measured by ImmunoCAP ≥ 1kU_A/_L. Subjects with a history or currently have clinically significant gastrointestinal, hepatic, renal, cardiovascular, endocrine, oncological, immunological, neurological, ophthalmological, haematological, respiratory or psychiatric disorder or any other condition, which in the opinion of the investigator or sponsor would jeopardize the safety of the subject or the validity of the study results, severe or uncontrolled asthma as assessed by the GINA Asthma symptom control questionnaire, subjects with a medical history of any previous episode of severe or life-threatening anaphylaxis or anaphylactic shock, subjects with skin disorders that would hinder skin testing and/or its interpretation (e.g., severe generalized active atopic dermatitis, subject that previously received therapy aimed at desensitising cat allergy, subjects that received allergen specific immunotherapy within the last 5 years or have received monoclonal antibody therapy in the last 12 months were all excluded from the study.

**PBMC Isolation**

Heparinized blood diluted 1:1 with RPMI 1640 media (Invitrogen; Thermo Fisher Scientific) was layered on 30% Ficoll-Paque Plus (GE Healthcare) density gradient and centrifuged for 25 minutes at 2200 rpm at room temperature. The PBMC layer was collected, washed, and resuspended in RPMI 1640. The cell viability was greater than 97%, as determined by trypan blue exclusion.

***In Vitro* T- and B-cell stimulation**

PBMCs were cultured for up to 6 days (T cells and regulatory T cells) or 72 hours (B cells) with varying concentrations of allergen extracts or Fel d1 eBP. For *in vitro* T-cell stimulation, PBMCs were stained with CellTrace Violet (Thermo Fisher Scientific) before stimulation with allergen extracts or bioparticle. Cells were washed with culture medium and stimulated with phorbol 12-myristate 13-acetate (50 ng/mL; Sigma- Aldrich, St Louis, Mo) and ionomycin (1 mg/mL; Sigma-Aldrich) in the presence of monensin (20 mg/mL; BioLegend, San Diego, Calif) or brefeldin A (1:10; BD Biosciences, San Jose, Calif) for 5 hours before staining. Cells were then blocked with Fc blocking agent (Miltenyi Biotec, San Diego, Calif). Cells were immunostained with cell-surface (CD5, CD19, CD38, CD24, CD4, CD161, CD27, CRTH2, CXCR5, ICOS, PD-1) and intracellular (IL-10, IL-4, IL-21) antibodies and acquired on the BD LSRFortessa device (BD Biosciences). Regulatory T cells were immediately blocked after 6 days of culture with Fc blocking agent (Miltenyi Biotec, San Diego, Calif) and cells were immunostained with cells surface antibodies (CD4, CD25, CD127, CD45RO) and intranuclear antibodies (CTLA-4, FoxP3, SATB1) following nuclear permeabilization using Fixation/Permeabilization Concentrate (1:4 eBiosciences™ Fixation/Perm Diluent; Thermo Fisher Scientific). Regulatory T cells were acquired on the BD LSRFortessa device (BD Biosciences).

**Unbiased clustering analysis**

Machine learning–driven unbiased clustering analyses (viSNE and FlowSOM) were performed on the flow cytometry data. Analysis using viSNE and FlowSOM was performed on a CD4 T-cell population or a CD19 B-cell population, and the cluster setting was set to markers CRTH2, CXCR5, PD-1, and CD27 or to markers CD5, CD38, CD27, and CD24, respectively. FCS files from multiple patients within the same group were concatenated to generate a representative data set (FCSConcat2). Red and blue in the viSNE map repre- sent high and low expression of the corresponding markers, respectively. FlowSOM analysis was performed on a predetermined metacluster setting of 20 or 14 (T and B cells, respectively). Star plots generated via FlowSOM allowed us to identify 2 pieces of information: first, the size of the cluster nodes representing population abundance; and second, the proportion and distance of the pie chart within each cluster node representing the expression of markers.

**Supplementary Figure Legends**

**Supplementary Figure 1. Fel d 1 *e*BP lacks the capacity to modulate T cell responses in non-atopic controls. (A-B)** Effect of response to increasing doses of natural Fel d 1 and Fel d 1 *e*BP *in vitro* in CA (n = 12) subjects on proliferation of *(A)* T_H_2 (CD4^+^, CD27^-^, CRTH2^+^), T_H_2A (CD4^+^, CD27^-^, CRTH2^+^, CD161^+^, CD49d^+^), Tfh cells (CD4^+^, CXCR5^+^, PD-1^+^) and *(B)* T_H_1 (CD4^+^, IFN𝛄^+^), IL10^+^ non-T_H_2 (CD4^+^,CRTH2^-^,IL10^+^), and IL10^+^ Treg (CD4^+^, CD25^+^, CD127^lo^, IL10^+^) cells. Between-group comparison statistical analysis was performed by Mann-Whitney U test; *P < .05, **P < .01, ***P < .001. Data are shown as means ± SEMs.

**Supplementary Figure 2. Fel d 1 *e*BP lacks the capacity to induce Natural, Memory and Naïve Treg subsets.** **(A)** Flow cytometry representative plots of Treg (CD4^+^CD25^hi^CD127^lo^) cell response to no stimulation, natural Fel d 1 or Fel d 1 *e*BP stimulation in CA and NAC subjects. **(B)** Effect of natural Fel d 1 and Fel d 1 *e*BP on Natural (CD4^+^FOXP3^+^CD25^hi^CD127^lo^), Memory (CD4^+^CD45RO^+^FOXP3^+^CD25^hi^CD127^lo^), Naïve (CD4^+^CD45RO^-^FOXP3^+^CD25^hi^ CD127^lo^) Treg cells of CA subjects (n=12) and NAC subjects (n=10). **(C)** Effect of natural Fel d 1 and Fel d 1 *e*BP on functional Natural (SATB1^-^FOXP3^+^CD25^hi^CD127^lo^), Memory (SATB1^-^CD45RO^+^FOXP3^+^CD25^hi^CD127^lo^), Naïve (SATB1^-^CD45RO^-^FOXP3^+^CD25^hi^CD127^lo^) Treg subsets of CA subjects (n=12) and NAC subjects (n=10). Between-group comparison statistical analysis was performed by Mann-Whitney U test; *P < .05, **P < .01, ***P < .001. Data are shown as means ± SEMs.

**Supplementary Figure 3. Differential gene expression analysis of monocytes treated with natural Fel d 1, Fel d 1 *e*BP and untreated. (A)** Volcano plot depiction and **(B)** heatmap representation of the top 30 differential expressed genes by adjusted p-value between natural Fel d 1 and Fel d 1 *e*BP (Left), unstimulated and natural Fel d 1 (middle), and unsimulated and Fel d 1 BP treated PBMCs performed using scRNAseq following 6 days of *in vitro* stimulation. Statistical analyses were performed using ANOVA with P<0.05 denoting a differential expression.

**Supplementary Figure 4. Single-cell RNA sequencing reveals 13 distinct clusters of monocytes. (A)**UMAP showing 13 distinct clusters of monocytes from PBMCs following 6 days of *in vitro* stimulation with natural Fel d 1, Fel d 1 *e*BP and unstimulated from 3 cat allergic individuals. **(B)** Heatmap depicting the expression of the top 5 genes in each of the monocyte clusters labelled g0-g12.

**Supplementary Figure 5. Differential abundance testing of scRNAseq data identifies a Fel d 1 *e*BP targeted neighbourhoods of naïve B cells. (A-B)** Beeswarm plots depicting the differential abundance of different neighbourhoods within the B cell subclusters and (B) highlighted neighbourhoods targeted by the Fel d 1 *e*BP (P-adjusted < 0.05).

**Supplementary Figure 6. Gene Ontology GSEA Pathway analysis of Treg cells reveals a dampening of type 2 inflammatory signalling pathway in the presence of Fel d 1 *e*BP.** Schematic representation of the clustering of gene ontology pathways determined by GSEA pathway enrichment analysis of Fel d 1 *e*BP treated Treg cells compared to natural Fel d 1, with highlighted pathways of interest and the associated genes and corresponding normalised enrichment score (NES; NES= +1.6, P<0.2 and NES= -1.76, P < 0.2)

**Supplementary Figure 7. Fel d 1 *e*BP demonstrates hypoallergenic characteristics *in vitro*. (A)** Flow cytometry representative plots of basophil activation and histamine release after *ex vivo* natural Fel d 1 or Fel d 1 *e*BP stimulation. **(B-D)** Effect of natural Fel d 1 and Fel d 1 *e*BP on *(B)* basophil activation (CD203c^bright^) of CA subjects (n = 12) and the effect of natural Fel d 1 on basophil activation of NAC subjects (n = 12), and *(C)* the corresponding AUC analysis of basophil activation and histamine release after natural Fel d1 and Fel d 1 *e*BP stimulation of CA subjects (n = 12). **(D)** Table demonstrating AUC of basophil activation and histamine release**.**
