# Supplementary figures and images for: Fel d 1‐Expressing Plant‐Derived Bioparticle: A Novel Treatment for Cat Allergy
Source: Allergy. 2026 Mar 19;81(6):2156–71. doi: 10.1111/all.70280 (PMC13256289; doi:10.1111/all.70280)

## Slide 1
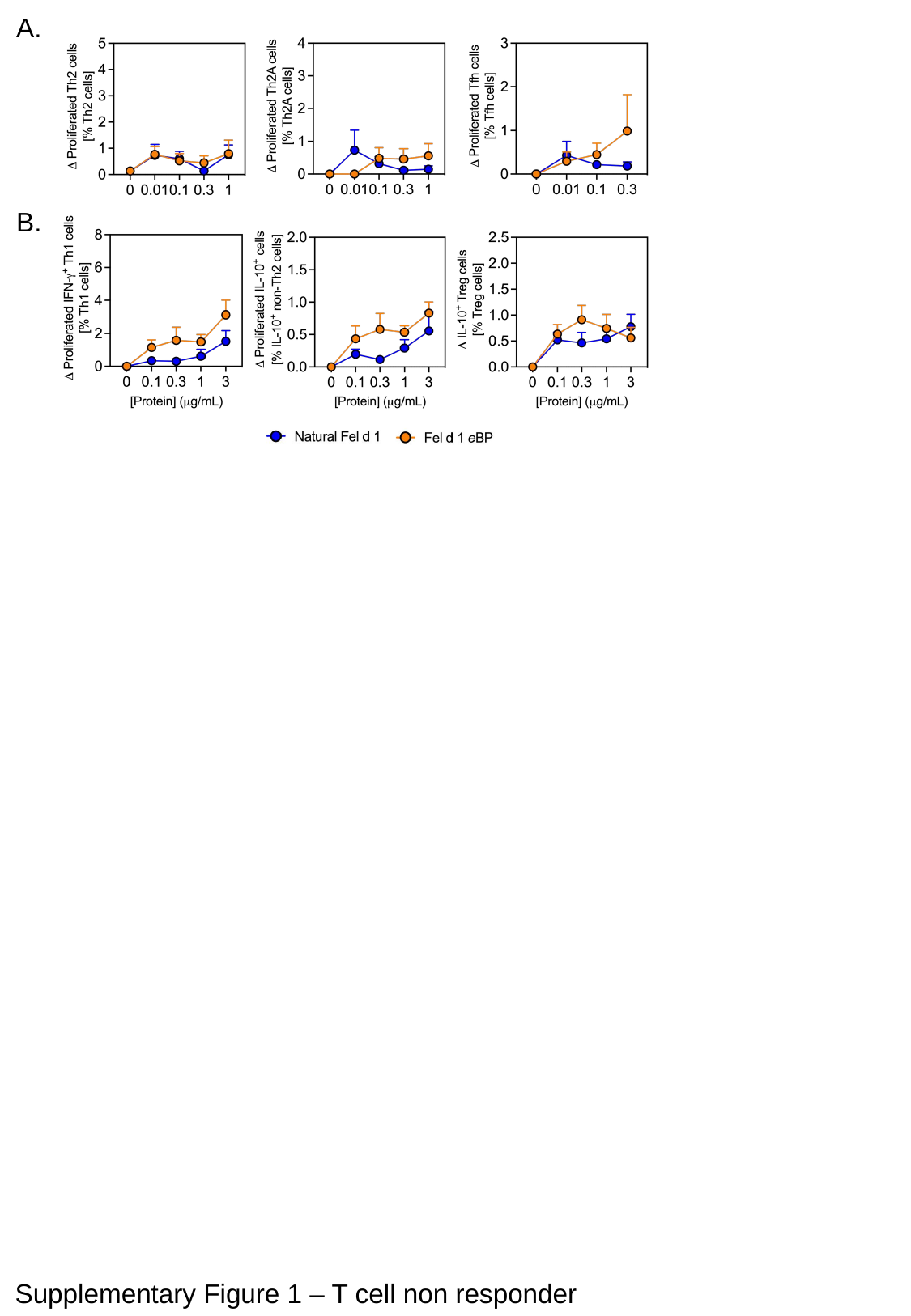

A.
B.
Supplementary Figure 1 – T cell non responder

Supplement: Supplementary file 2 — Figure S1 Fel d 1 eBP lacks the capacity to modulate T cell responses in non‐atopic controls. (A, B) Effect of response to increasing doses of natural Fel d 1 and Fel d 1 eBP in vitro in CA (n = 12) subjects on proliferation of (A) TH2 (CD4+, CD27−, CRTH2+), TH2A (CD4+, CD27−, CRTH2+, CD161+, CD49d+), Tfh cells (CD4+, CXCR5+, PD‐1+) and (B) TH1 (CD4+, IFN𝛄+), IL10+ non‐TH2 (CD4+, CRTH2−, IL10+), and IL10+ Treg (CD4+, CD25+, CD127lo, IL10+) cells. Between‐group comparison statistical analysis was performed by the Mann–Whitney U test; *p < 0.05, **p < 0.01, ***p < 0.001. Data are shown as means ± SEMs. [file ALL-81-2156-s005.pptx]

## Slide 1
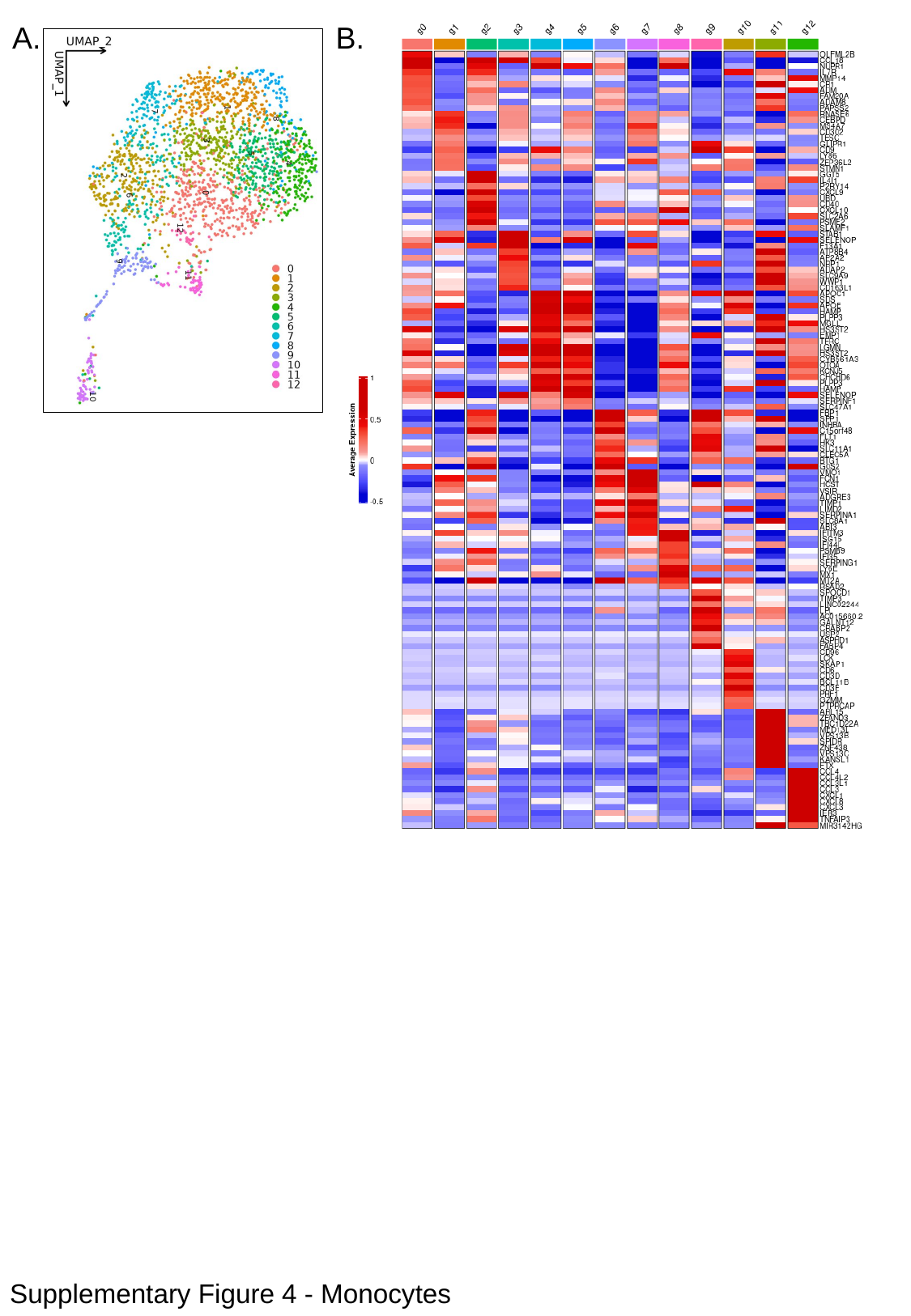

A.
B.
Supplementary Figure 4 - Monocytes

Supplement: Supplementary file 5 — Figure S4 Single‐cell RNA sequencing reveals 13 distinct clusters of monocytes. (A) UMAP showing 13 distinct clusters of monocytes from PBMCs following 6 days of in vitro stimulation with natural Fel d 1, Fel d 1 eBP and unstimulated from 3 cat‐allergic individuals. (B) Heatmap depicting the expression of the top 5 genes in each of the monocyte clusters labelled g0–g12. [file ALL-81-2156-s001.pptx]

## Slide 1
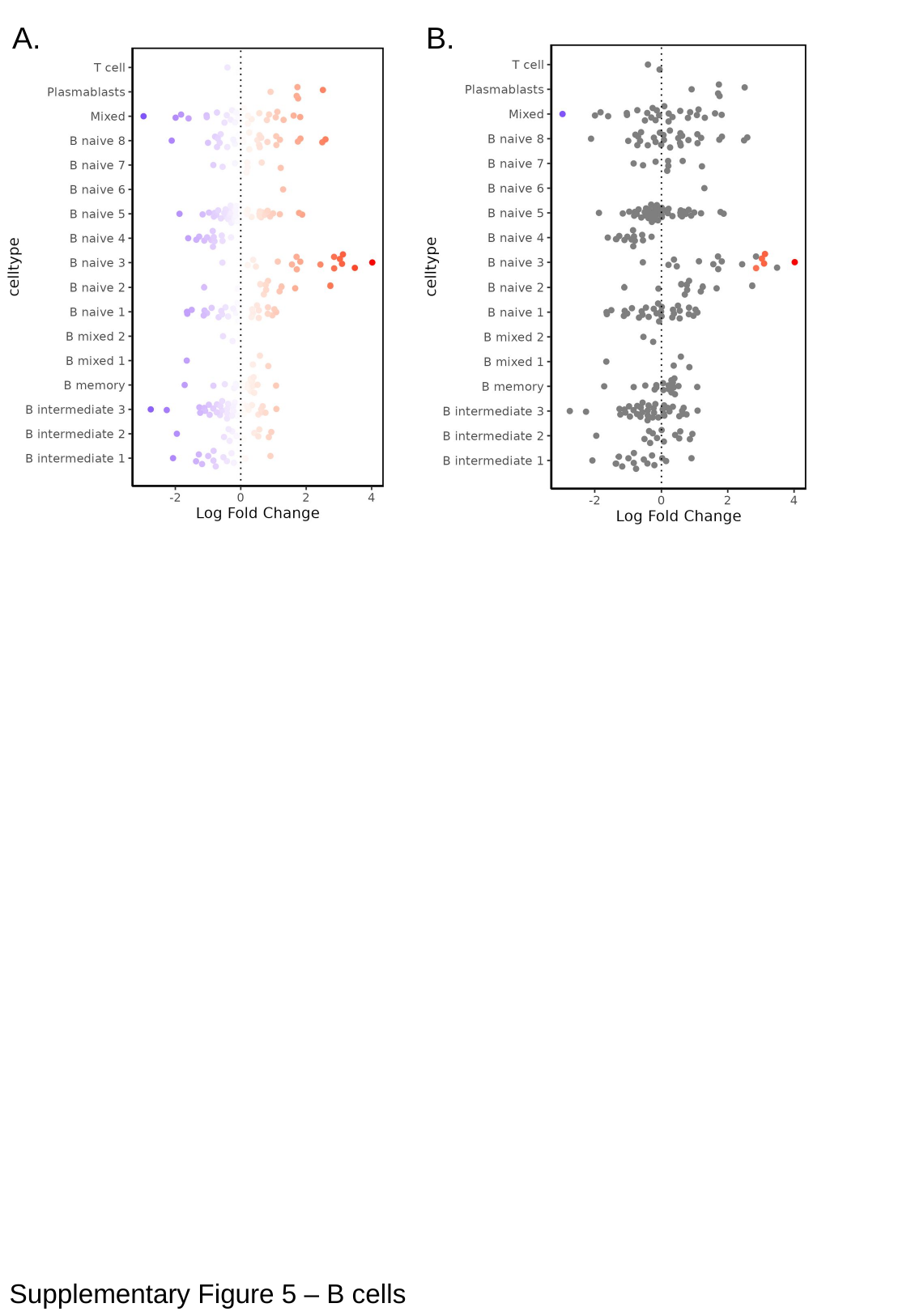

A.
B.
Supplementary Figure 5 – B cells

Supplement: Supplementary file 6 — Figure S5 Differential abundance testing of scRNAseq data identifies a Fel d 1 eBP targeted neighbourhoods of naïve B cells. (A, B) Beeswarm plots depicting the differential abundance of different neighbourhoods within the B cell subclusters and (B) highlighted neighbourhoods targeted by the Fel d 1 eBP (p ‐adjusted< 0.05). [file ALL-81-2156-s006.pptx]
