# Supplementary material for: Fel d 1‐Expressing Plant‐Derived Bioparticle: A Novel Treatment for Cat Allergy
Source: Allergy. 2026 Mar 19;81(6):2156–71. doi: 10.1111/all.70280 (PMC13256289; doi:10.1111/all.70280)
Supplement: Supplementary file 3 — Figure S2 Fel d 1 eBP lacks the capacity to induce Natural, Memory and Naïve Treg subsets. (A) Flow cytometry representative plots of Treg (CD4+CD25hiCD127lo) cell response to no stimulation, natural Fel d 1 or Fel d 1 eBP stimulation in CA and NAC subjects. (B) Effect of natural Fel d 1 and Fel d 1 eBP on Natural (CD4+FOXP3+CD25hiCD127lo), Memory (CD4+CD45RO+FOXP3+CD25hiCD127lo), Naïve (CD4+CD45RO−FOXP3+CD25hi CD127lo) Treg cells of CA subjects (n = 12) and NAC subjects (n = 10). (C) Effect of natural Fel d 1 and Fel d 1 eBP on functional Natural (SATB1−FOXP3+CD25hiCD127lo), Memory (SATB1−CD45RO+FOXP3+CD25hiCD127lo), Naïve (SATB1−CD45RO−FOXP3+CD25hiCD127lo) Treg subsets of CA subjects (n = 12) and NAC subjects (n = 10). Between‐group comparison statistical analysis was performed by the Mann–Whitney U test; *p < 0.05, **p < 0.01, ***p < 0.001. Data are shown as means ± SEMs. [file ALL-81-2156-s009.pptx]

## Slide 1
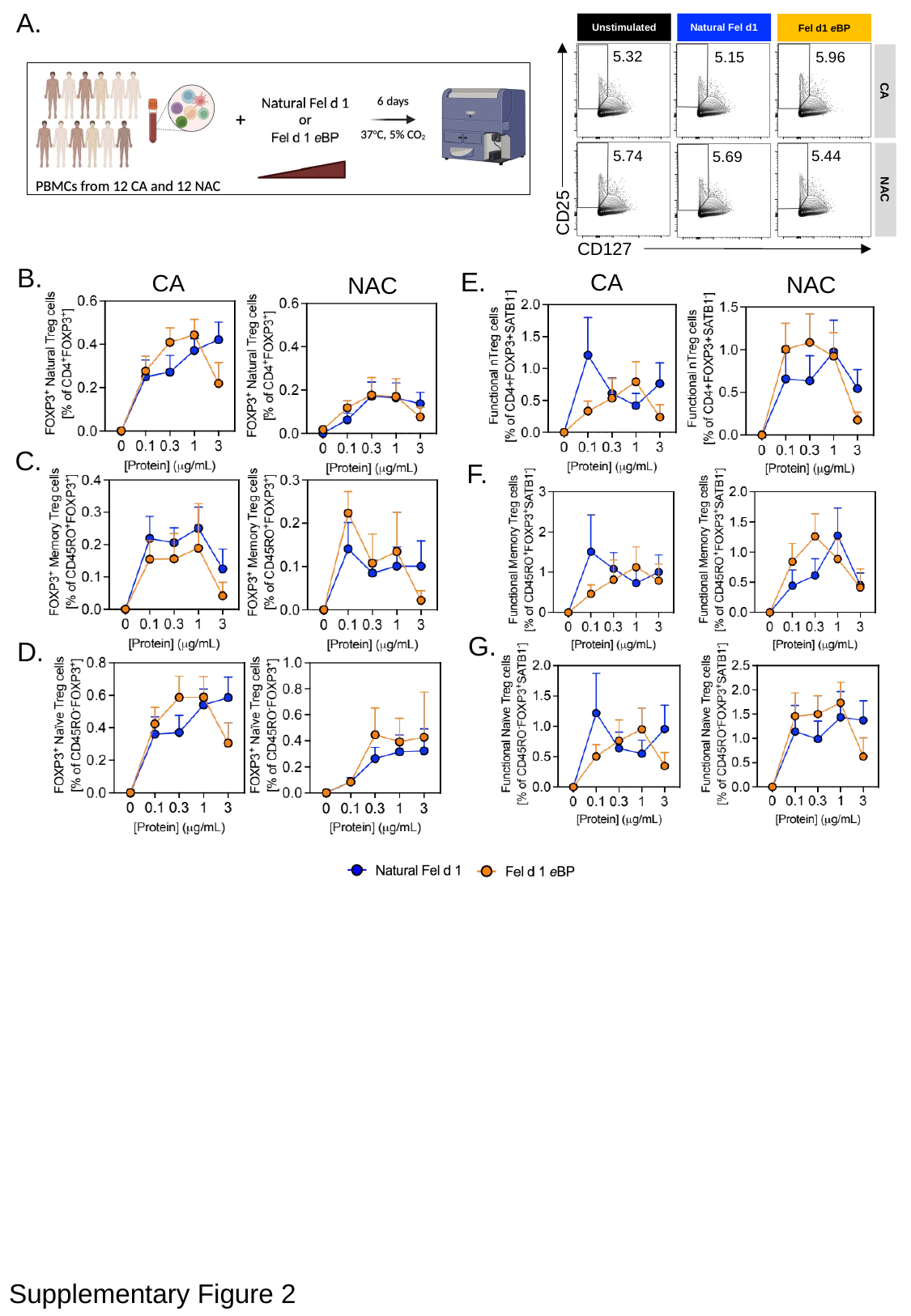

A.
Unstimulated
Natural Fel d1
Fel d1 eBP
5.32
5.15
5.96
5.44
5.74
5.69
CA
NAC
CD25
CD127
B.
E.
CA
CA
NAC
NAC
C.
F.
G.
D.
Supplementary Figure 2
