# Supplementary material for: Fel d 1‐Expressing Plant‐Derived Bioparticle: A Novel Treatment for Cat Allergy
Source: Allergy. 2026 Mar 19;81(6):2156–71. doi: 10.1111/all.70280 (PMC13256289; doi:10.1111/all.70280)
Supplement: Supplementary file 4 — Figure S3 Differential gene expression analysis of monocytes treated with natural Fel d 1, Fel d 1 eBP and untreated. (A) Volcano plot depiction and (B) heatmap representation of the top 30 differentially expressed genes by adjusted p‐value between natural Fel d 1 and Fel d 1 eBP (Left), unstimulated and natural Fel d 1 (middle), and unstimulated and Fel d 1 BP‐treated PBMCs performed using scRNAseq following 6 days of in vitro stimulation. Statistical analyses were performed using ANOVA, with p < 0.05 denoting a differential expression. [file ALL-81-2156-s007.pptx]

## Slide 1
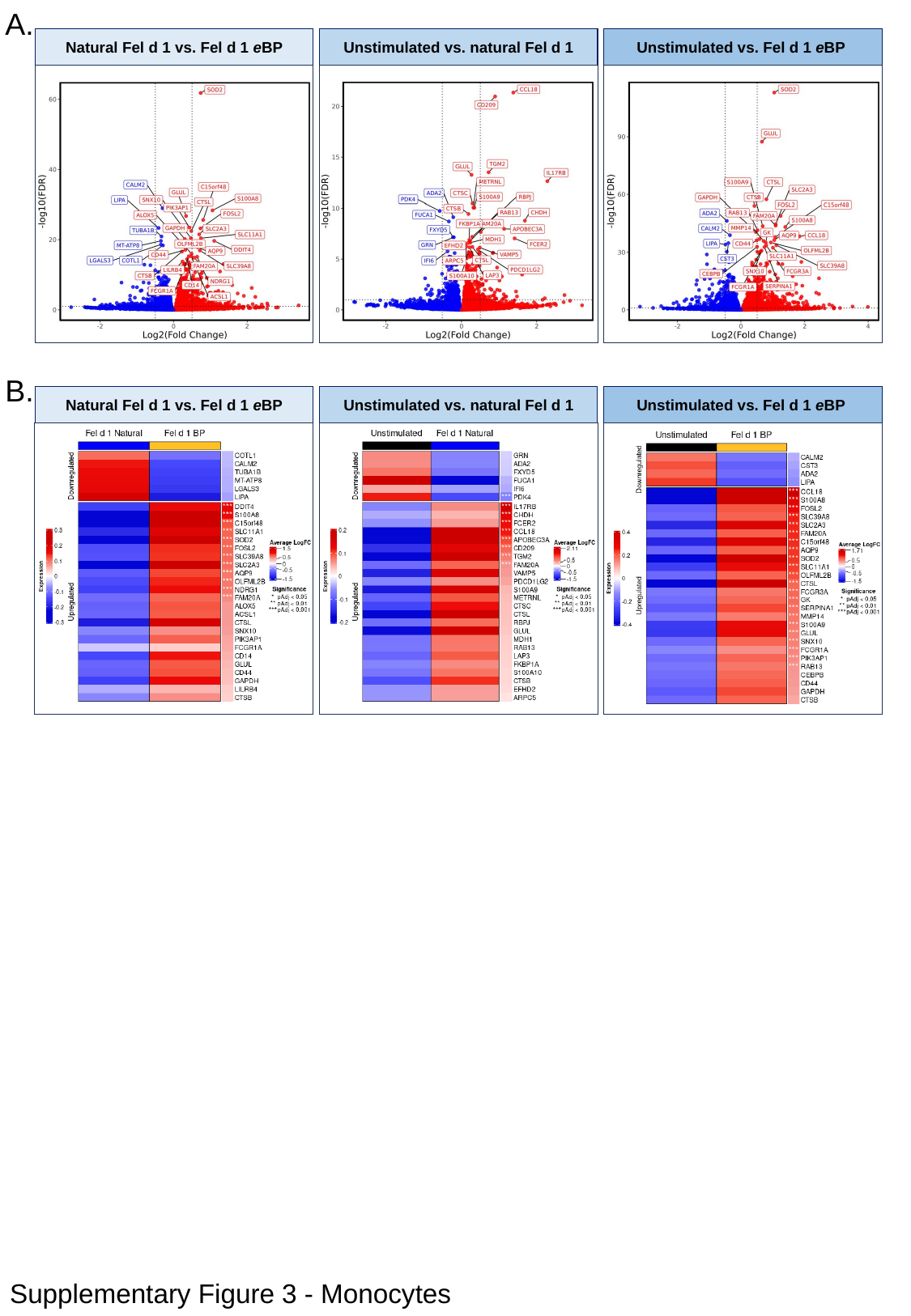

A.
Natural Fel d 1 vs. Fel d 1 eBP
Unstimulated vs. natural Fel d 1
Unstimulated vs. Fel d 1 eBP
B.
Natural Fel d 1 vs. Fel d 1 eBP
Unstimulated vs. natural Fel d 1
Unstimulated vs. Fel d 1 eBP
Supplementary Figure 3 - Monocytes
