# Supplementary material for: Fel d 1‐Expressing Plant‐Derived Bioparticle: A Novel Treatment for Cat Allergy
Source: Allergy. 2026 Mar 19;81(6):2156–71. doi: 10.1111/all.70280 (PMC13256289; doi:10.1111/all.70280)
Supplement: Supplementary file 7 — Figure S6 Gene Ontology GSEA Pathway analysis of Treg cells reveals a dampening of type 2 inflammatory signalling pathway in the presence of Fel d 1 eBP. Schematic representation of the clustering of gene ontology pathways determined by GSEA pathway enrichment analysis of Fel d 1 eBP‐treated Treg cells compared to natural Fel d 1, with highlighted pathways of interest and the associated genes and corresponding normalised enrichment score (NES; NES = +1.6, p < 0.2 and NES = −1.76, p < 0.2). [file ALL-81-2156-s003.pptx]

## Slide 1
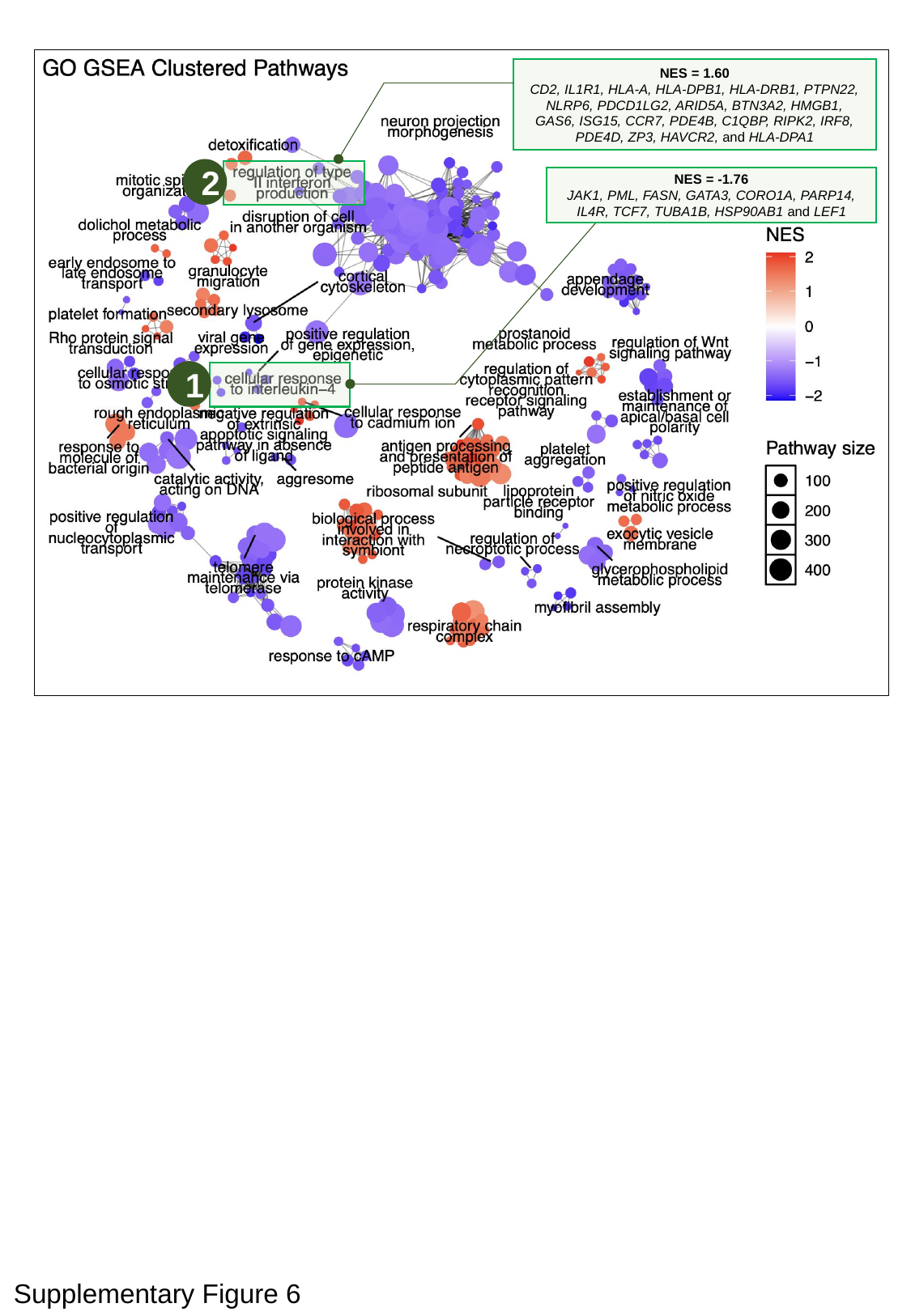

NES = 1.60
CD2, IL1R1, HLA-A, HLA-DPB1, HLA-DRB1, PTPN22, NLRP6, PDCD1LG2, ARID5A, BTN3A2, HMGB1, GAS6, ISG15, CCR7, PDE4B, C1QBP, RIPK2, IRF8, PDE4D, ZP3, HAVCR2, and HLA-DPA1
2
NES = -1.76
JAK1, PML, FASN, GATA3, CORO1A, PARP14, IL4R, TCF7, TUBA1B, HSP90AB1 and LEF1
1
Supplementary Figure 6
