# Supplementary material for: Fel d 1‐Expressing Plant‐Derived Bioparticle: A Novel Treatment for Cat Allergy
Source: Allergy. 2026 Mar 19;81(6):2156–71. doi: 10.1111/all.70280 (PMC13256289; doi:10.1111/all.70280)
Supplement: Supplementary file 8 — Figure S7Fel d 1 eBP demonstrates hypoallergenic characteristics in vitro. (A) Flow cytometry representative plots of basophil activation and histamine release after ex vivo natural Fel d 1 or Fel d 1 eBP stimulation. (B–D) Effect of natural Fel d 1 and Fel d 1 eBP on (B) basophil activation (CD203cbright) of CA subjects (n = 12) and the effect of natural Fel d 1 on basophil activation of NAC subjects (n = 12), and (C) the corresponding AUC analysis of basophil activation and histamine release after natural Fel d1 and Fel d 1 eBP stimulation of CA subjects (n = 12). (D) Table demonstrating the AUC of basophil activation and histamine release. [file ALL-81-2156-s004.pptx]

## Slide 1
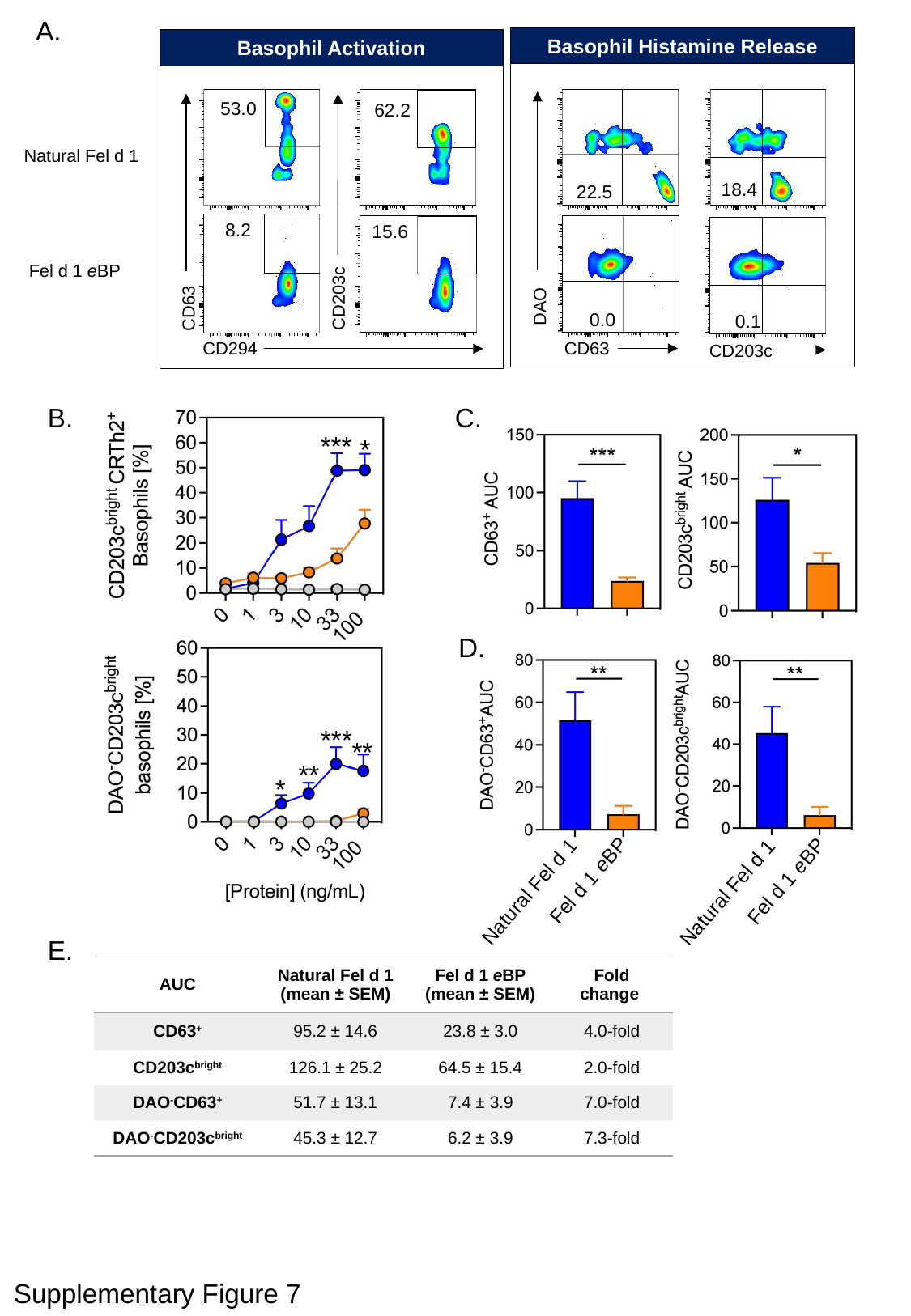

A.
Basophil Histamine Release
Basophil Activation
53.0
62.2
Natural Fel d 1
18.4
22.5
8.2
15.6
Fel d 1 eBP
CD203c
DAO
CD63
0.0
0.1
CD294
CD63
CD203c
Fel d 1 eBP
Natural Fel d 1
B.
C.
***
*
D.
***
**
**
*
Fel d 1 eBP
Natural Fel d 1
E.
| AUC | Natural Fel d 1 (mean ± SEM) | Fel d 1 eBP (mean ± SEM) | Fold change |
| --- | --- | --- | --- |
| CD63+ | 95.2 ± 14.6 | 23.8 ± 3.0 | 4.0-fold |
| CD203cbright | 126.1 ± 25.2 | 64.5 ± 15.4 | 2.0-fold |
| DAO-CD63+ | 51.7 ± 13.1 | 7.4 ± 3.9 | 7.0-fold |
| DAO-CD203cbright | 45.3 ± 12.7 | 6.2 ± 3.9 | 7.3-fold |
Supplementary Figure 7
