# Supplementary material for: Fel d 1‐Expressing Plant‐Derived Bioparticle: A Novel Treatment for Cat Allergy
Source: Allergy. 2026 Mar 19;81(6):2156–71. doi: 10.1111/all.70280 (PMC13256289; doi:10.1111/all.70280)
Supplement: Supplementary file 9 — Table S1 Baseline skin wheal diameter statistics. Table S2 Mean post‐SPT wheal size following ANG101 and ALK Soluprick administration. Highlighted values are associated with patients showing a minimal 5 mm wheal diameter for both products. [file ALL-81-2156-s002.docx]

Supplementary Table 1. Baseline skin wheal diameter statistics

|  | | **ELIGIBLE PATIENTS** | | |
| --- | --- | --- | --- | --- |
|  |  | **First Cat dander** | **Second Cat dander** | **Mean** |
| **WHEAL DIAMETER** | **Valid N** | 20 | 20 | 20 |
|  | **Mean** | 8.90 | 8.48 | 8.69 |
|  | **Standard Deviation** | 1.74 | 1.52 | 1.44 |
|  | **95.0% Lower CL for Mean** | 8.09 | 7.76 | 8.01 |
|  | **95.0% Upper CL for Mean** | 9.71 | 9.19 | 9.36 |
|  | **Standard Error of Mean** | .39 | .34 | .32 |
|  | **Median** | 8.50 | 8.00 | 8.50 |
|  | **Minimum** | 7.00 | 7.00 | 7.00 |
|  | **Maximum** | 13.00 | 12.00 | 12.50 |

**Supplementary Table 2. Mean post SPT wheal size following ANG101 and ALK Soluprick administration.** Highlighted values are associated with patients showing a minimal 5 mm wheal diameter for both products.

| **Subject** | **Wheal Size** | | | | | | | | | | | | | | | |
| --- | --- | --- | --- | --- | --- | --- | --- | --- | --- | --- | --- | --- | --- | --- | --- | --- |
|  | **TEST** | | | | | | | | | | | | | | | |
|  | **ALK** | | | | | | | | **ANG101** | | | | | | | |
|  | **Dilution** | | | | | | | | **Dilution** | | | | | | | |
|  | **1/1.2** | **1/6** | **1/60** | **1/600** | **1/6000** | **1/60000** | **Diluent (saline)** | **Histamine 10mg/ml** | **1/5** | **1/50** | **1/500** | **1/5000** | **1/50000** | **Diluent (saline)** | **Histamine 10mg/ml** | **Undiluted** |
| 1 | 6.0 | 5.0 | 5.0 | 2.0 | 0.0 | 0.0 | 0.0 | 7.0 | 0.0 | 0.0 | 0.0 | 0.0 | 0.0 | 0.0 | 10.0 | 5.0 |
| 3 | 15.0 | 12.0 | 5.0 | 2.0 | 0.0 | 0.0 | 0.0 | 4.0 | 1.0 | 0.0 | 0.0 | 0.0 | 0.0 | 0.0 | 4.0 | 2.0 |
| 4 | 7.0 | 5.0 | 4.0 | 2.0 | 2.0 | 0.0 | 0.0 | 10.0 | 0.0 | 0.0 | 0.0 | 0.0 | 0.0 | 0.0 | 9.0 | 3.0 |
| 6 | 8.0 | 7.0 | 5.0 | 3.0 | 0.0 | 0.0 | 0.0 | 7.0 | 3.0 | 1.0 | 1.0 | 0.0 | 0.0 | 0.0 | 6.0 | 5.0 |
| 9 | 9.0 | 7.0 | 5.0 | 4.0 | 0.0 | 0.0 | 0.0 | 10.0 | 3.0 | 3.0 | 0.0 | 0.0 | 0.0 | 0.0 | 7.0 | 4.0 |
| 11 | 8.0 | 7.0 | 6.0 | 1.0 | 0.0 | 0.0 | 0.0 | 7.0 | 2.0 | 1.0 | 1.0 | 1.0 | 0.0 | 0.0 | 7.0 | 4.0 |
| 14 | 12.0 | 8.0 | 8.0 | 5.0 | 4.0 | 0.0 | 0.0 | 6.0 | 4.0 | 3.0 | 3.0 | 2.0 | 2.0 | 0.0 | 7.0 | 5.0 |
| 20 | 7.0 | 5.0 | 3.0 | 2.0 | 0.0 | 0.0 | 0.0 | 7.0 | 2.0 | 2.0 | 0.0 | 0.0 | 0.0 | 0.0 | 7.0 | 3.0 |
| 23 | 7.0 | 6.0 | 4.0 | 0.0 | 0.0 | 0.0 | 0.0 | 8.0 | 3.0 | 3.0 | 0.0 | 0.0 | 0.0 | 0.0 | 6.0 | 4.0 |
| 25 | 7.0 | 6.0 | 4.0 | 2.0 | 0.0 | 0.0 | 0.0 | 5.0 | 3.0 | 2.0 | 0.0 | 0.0 | 0.0 | 0.0 | 5.0 | 3.5 |
| 29 | 10.0 | 9.0 | 7.0 | 6.0 | 5.0 | 0.0 | 0.0 | 10.0 | 2.0 | 1.0 | 0.0 | 0.0 | 0.0 | 0.0 | 8.0 | 2.0 |
| 33 | 13.0 | 7.0 | 4.0 | 2.0 | 0.0 | 0.0 | 0.0 | 6.0 | 0.0 | 0.0 | 0.0 | 0.0 | 0.0 | 0.0 | 5.0 | 3.0 |
| 34 | 7.0 | 6.0 | 5.0 | 4.0 | 3.0 | 0.0 | 0.0 | 4.5 | 4.0 | 0.0 | 0.0 | 0.0 | 0.0 | 0.0 | 5.0 | 5.0 |
| 35 | 8.5 | 6.0 | 5.5 | 4.0 | 0.0 | 0.0 | 0.0 | 4.0 | 2.0 | 0.0 | 0.0 | 0.0 | 0.0 | 0.0 | 5.0 | 5.0 |
| 36 | 7.0 | 7.0 | 4.0 | 2.0 | 0.0 | 0.0 | 0.0 | 7.0 | 3.0 | 2.0 | 0.0 | 0.0 | 0.0 | 0.0 | 5.0 | 4.0 |
| 37 | 6.0 | 6.0 | 4.0 | 0.0 | 0.0 | 0.0 | 0.0 | 5.0 | 0.0 | 0.0 | 0.0 | 0.0 | 0.0 | 0.0 | 4.0 | 3.0 |
| 38 | 8.0 | 8.0 | 6.0 | 4.0 | 3.0 | 2.0 | 0.0 | N/A | 4.0 | 3.0 | 0.0 | 0.0 | 0.0 | 0.0 | 7.0 | 6.0 |
| 39 | 9.0 | 8.0 | 5.0 | 4.0 | 0.0 | 0.0 | 0.0 | 8.0 | 4.0 | 3.0 | 0.0 | 0.0 | 0.0 | 0.0 | 11.0 | 5.0 |
| 41 | 5.0 | 5.0 | 2.0 | 2.0 | 0.0 | 0.0 | 0.0 | 8.0 | 2.0 | 1.0 | 0.0 | 0.0 | 0.0 | 0.0 | 8.0 | 3.0 |
| 44 | 7.0 | 5.0 | 4.0 | 2.0 | 0.0 | 0.0 | 0.0 | 8.0 | 3.0 | 2.0 | 0.0 | 0.0 | 0.0 | 0.0 | 8.0 | 4.0 |
